# Redundancy and the Evolution of *Cis*-Regulatory Element Multiplicity

**DOI:** 10.1371/journal.pcbi.1000848

**Published:** 2010-07-08

**Authors:** Tiago Paixão, Ricardo B. R. Azevedo

**Affiliations:** Department of Biology and Biochemistry, University of Houston, Houston, Texas, United States of America; University of California San Diego, United States of America

## Abstract

The promoter regions of many genes contain multiple binding sites for the same transcription factor (TF). One possibility is that this multiplicity evolved through transitional forms showing redundant *cis*-regulation. To evaluate this hypothesis, we must disentangle the relative contributions of different evolutionary mechanisms to the evolution of binding site multiplicity. Here, we attempt to do this using a model of binding site evolution. Our model considers binding sequences and their interactions with TFs explicitly, and allows us to cast the evolution of gene networks into a neutral network framework. We then test some of the model's predictions using data from yeast. Analysis of the model suggested three candidate nonadaptive processes favoring the evolution of *cis*-regulatory element redundancy and multiplicity: neutral evolution in long promoters, recombination and TF promiscuity. We find that recombination rate is positively associated with binding site multiplicity in yeast. Our model also indicated that weak direct selection for multiplicity (partial redundancy) can play a major role in organisms with large populations. Our data suggest that selection for changes in gene expression level may have contributed to the evolution of multiple binding sites in yeast. We conclude that the evolution of *cis*-regulatory element redundancy and multiplicity is impacted by many aspects of the biology of an organism: both adaptive and nonadaptive processes, both changes in *cis* to binding sites and in trans to the TFs that interact with them, both the functional setting of the promoter and the population genetic context of the individuals carrying them.

## Author Summary

TFs regulate gene expression by binding to specific sequences in the promoter regions of their target genes. Promoters often contain multiple copies of the same TF binding sites. How does this multiplicity evolve? One possibility is that individuals with multiple, redundant binding sites have higher fitness. However, nonadaptive processes are also likely to be important. Here, we develop a mathematical model of the evolution of TF binding sites to help us disentangle how different evolutionary mechanisms contribute to the evolution of binding site redundancy and multiplicity. We show that recombination is expected to promote the evolution of multiple binding sites. This prediction is corroborated by genome-wide data from yeast. Another important factor in the evolution of multiplicity predicted in our analysis is TF promiscuity, that is, the ability of a TF to bind to multiple sequences. In addition, our analysis indicated that direct selection can have large effects on the evolution of redundancy and multiplicity. Data from yeast identified selection for changes in expression level as a candidate mechanism for the evolution of multiple binding sites. We conclude that, although selection may play a major role in the evolution of multiplicity in regulatory regions, nonadaptive forces can also lead to high levels of multiplicity.

## Introduction

Promoters frequently contain multiple functional regulatory elements [1]. For example, the regulatory region for stripe 2 of *even-skipped* (*eve*) of the fruit fly *Drosophila melanogaster* comprises 17 binding sites for four transcription factors (TFs), including five binding sites (B1–B5) for the activator *bicoid* (*bcd*) [2]. How does *cis*-regulatory element multiplicity evolve? There are three possibilities. First, perhaps “more is better” when it comes to TF binding sites. Multiple binding sites may cause changes in the level of gene expression or in its robustness against variation in TF concentrations [1], [3]–[5]. Second, multiplicity might be favored by selection, but independently of its functional consequences. For example, genotypes with many binding sites may be more likely to produce viable offspring after mutation or recombination with genotypes with fewer binding sites [6]–[9]. Third, *cis*-regulatory element multiplicity may arise by nonadaptive processes [9]–[11]. Stone and Wray [10] have shown that a population of 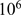 diploid individuals could evolve two identical copies of a 6 base pair (bp) binding site in a 200-bp promoter every 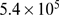 generations through random mutation and genetic drift alone. The intergenic regions of *Saccharomyces cerevisiae* are 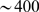 bp long on average, whereas those of multicellular eukaryotes can be orders of magnitude longer.

**Figure 1:**
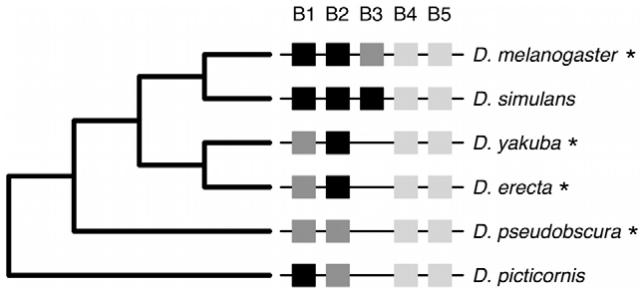
Evolution of the *bcd* binding sites in the *eve* stripe 2 enhancer in *Drosophila*. Phylogenetic relationships among 6 species of *Drosophila* [69] and *bcd* binding sites in their stripe 2 enhancers [3]. Squares represent the five binding sites (B1–B5) found in different species. The darkness of the square represents the closeness of the match between the binding site and the consensus *bcd* recognition sequence [30]: black, 8/9 nucleotides; dark gray, 7/9; light gray, 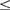
6/9. The stripe 2 enhancers of species marked with an asterisk were able to drive reporter gene expression in *D. melanogaster* embryos coincident with native *eve* stripe 2 [3].

The common thread to all the evolutionary scenarios listed above is redundancy, the ability of structurally identical elements to contribute to the same function [12]–[16]. Redundancy is thought to be widespread in biological systems. In eukaryotes, a large proportion of genes are duplicates, and deletion of one copy often has little or no phenotypic effect because the other copy can compensate for the loss of function [17]. Functionality and redundancy are more difficult to establish for the case of multiple *cis*-regulatory elements [1]. The five *bcd* binding sites in *eve* the stripe 2 enhancer are not fully redundant because loss-of-function mutations to B1, B2 or B3 cause reduced *eve* stripe 2 expression and gain-of-function mutations to B4 and B5 lead to increased expression [2], [18]. However, redundancy was likely important in the evolution of these sites. When Ludwig and colleagues [3] compared the stripe 2 enhancers of different species of *Drosophila*, they found that some of them lacked the B3 site (Figure 1). This observation implies that the B3 site evolved recently in the lineage leading to the last common ancestor of *D. melanogaster* and *D. simulans*. Furthermore, the B3 site was probably redundant when it first appeared because the stripe 2 enhancers of three species lacking the B3 binding site were able to drive expression of a reporter gene in *D. melanogaster* embryos coincident with native *eve* stripe 2 (Figure 1). Thus, redundant transitional forms can, in principle, play an important role in the evolution of *cis*-regulatory element multiplicity [1], [19]. In this paper we develop a model of binding site evolution and use it to evaluate the plausibility of different scenarios for the evolution of *cis*-regulatory element redundancy and multiplicity. We then test predictions obtained from our model using data from yeast.

### Model

Here we introduce a model of binding site evolution. The model extends earlier phenomenological models of the evolution of *cis*-regulatory element redundancy [7], [9], [11], [13], [16] in that it considers binding sequences and their interactions with TFs explicitly, albeit in a simplified manner [4]. We also build upon recent attempts to apply the mutational network approach [20], [21] to the study of gene regulatory networks [22]–[24]. We then use our model to investigate the conditions favoring the evolution of multiple TF binding sites.

### Gene regulation

A target gene has a promoter containing *cis*-regulatory sites for a number of TFs. 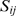 denotes the *j*th binding site for 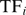. The TFs regulate expression of the target gene according to the following rules:

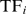
 binds preferentially to a canonical sequence 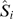 of length *n*.
2. The effect of a transcriptional activator 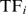 on target gene expression through the *j*th binding site is given by 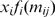, where 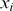 is the expression level of 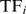, and 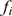 is a monotonically decreasing function of the number of mismatches 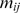 between 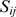 and 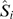 (i.e., the Hamming distance between the sequences), such that 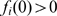 and 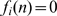. See Figure S1 for examples of *f* functions.
3. If 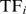 is a repressor then 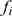 is a monotonically increasing function of *m*, such that 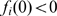 and 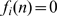.
4. The total effect of 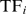 on gene expression is given by:
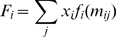
(1)

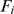
 will be positive for a transcriptional activator, and negative for a repressor.
5. Target gene activity is a monotonically increasing function of 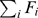.

Rules #2 and #3 are compatible with the two-state model for TF binding [4], [25]–[27]. Unless otherwise stated, our model deals with the evolution of the binding sites for a single transcriptional activator. For a discussion of how our model can be extended to repressors see ‘Generalizations and caveats’.

### Functionality, multiplicity and redundancy

The target gene is considered functional if, given normal levels of expression of its transcriptional regulators 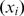, it is active above a threshold level, arbitrarily set to 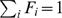 in this paper. Consider a promoter that contains 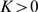 binding sites for 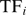 and is capable of sustaining gene function. A particular site 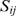 is considered functional if binding to the site has an effect on gene expression, that is, if 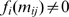. *Multiple* binding sites (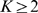) are considered *redundant* if at least one of them can be deleted without affecting gene function. *Full* redundancy occurs when the viability of redundant and nonredundant genotypes is the same; *partial* redundancy occurs when the viability of redundant genotypes is higher than that of nonredundant ones [12], [15] (see also ‘Natural selection’ below). Note that, according to the above definitions, multiplicity does not imply redundancy (full or partial).

### Mutation

In our model, the total effect of 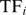 on the expression of a gene (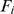, Equation 1) can change in three ways: a mutation in a binding site *j* that alters its 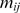 (*cis*), a mutation in the coding sequence of 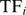 that modifies the 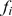 function directly (trans), and a change in the concentration of 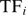, 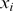. In the rest of the paper we consider only the first two types of evolution. We begin by considering the *cis* evolution of a single binding site.

**Figure 2:**
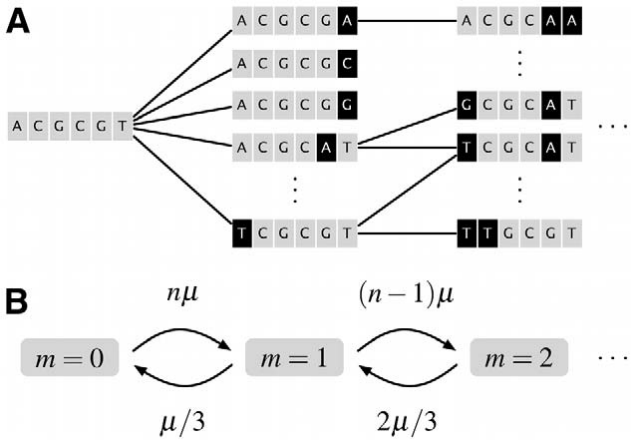
Condensed mutational network for a single binding site. (A) Mutational network for the Mbp1 canonical binding site [28]. Two sequences are connected if they differ by one base pair and the probability of any of these transitions is one-third of the mutation rate per base pair per generation (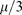). (B) Condensed version of the mutational network in (A). Genotypic classes were obtained by grouping all sequences at the same Hamming distance (*m*) from the canonical sequence. The probabilities of moving between genotypic classes are shown (*n* is the length of the binding site).

One way to represent the evolution of a binding site is through its mutational network [21]. Two genotypes are connected in a mutational network if one genotype can be obtained from the other through a single mutation. For example, the sequences ACGCGC and ACGCAT are both connected to ACGCGT, but not to each other, in the mutational network of all possible DNA sequences of length 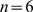 base pairs (Figure 2A). If the mutation rate per base pair per generation is 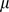, then ACGCGT will mutate into ACGCGC with a probability 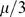. One difficulty with this approach is that even the relatively short sequences of TF binding sites (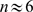 to 10 bp) define large mutational networks (e.g., the network of DNA sequences of length 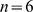 has 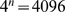 sequences).

Given that binding site functionality in our model is determined by the number of mismatches *m* relative to the canonical sequence, we can simplify the mutational network of a binding site by collapsing all sequences with a given *m*. This grouping of genotypes is appropriate because every sequence contained within a given *m* class has exactly the same number of mutational neighbors, both within the *m* class (obtained by mutating already mismatched sites) and in the neighboring 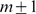 classes. A similar approach has been employed by others [4], [27]. We call the resulting network a *condensed* mutational network (Figure 2B). The *m* condensed genotypic class includes 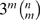 sequences. For example, if ACGCGT is the canonical sequence of the yeast TF Mbp1 [28], then both ACGCGC and ACGCAT belong to the 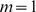 condensed class. The condensed mutational network representation is extremely compact, implying a reduction from 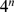 to 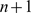 states for a binding site of length *n*.

Now we introduce evolution over the condensed mutational network. Consider an infinite-sized population of asexual, haploid organisms. Each individual has a genotype at a binding site of length *n*, such that the population is distributed over a condensed mutational network. The state of the population is given by a vector of frequencies 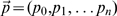, where 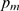 is the proportion of sequences in the population a Hamming distance *m* away from the canonical sequence. The population reproduces asexually, in discrete generations. Mutation causes the population to evolve according to the equation:
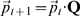
(2)where 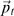 is the state of the population at time *t*, and **Q** is the transition matrix such that 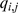 is the probability that the offspring from an individual with *i* mismatches has *j* mismatches. Assuming that a sequence cannot acquire more than one mutation in a single generation (appropriate for realistic values of 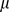), the nonzero elements of row *i* of **Q** are given by: 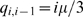, 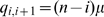 and 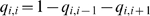. For example, the probability that the sequence ACGCGC from the 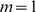 condensed class will mutate into the canonical sequence ACGCGT (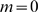) is 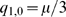. The probability that it will mutate into a sequence from class 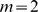 is the probability that a mutation occurs at a site other than the already mismatched site: 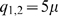. And the probability that it will remain in the 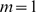 class is the probability that either no mutation occurs or that a mutation occurs at the mismatched site but does not result in the canonical sequence (C
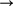
A or C
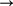
G, but not C
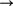
T): 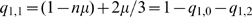. The transition probabilities are the same for any other 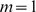 sequence, such as ACGCAT.

### Natural selection

We introduce selection by assuming that target gene function is required for viability. The population can only occupy states within the viable portion of the condensed mutational network. Every generation, mutant genotypes may appear in the *inviable* part of the condensed mutational network, but they fail to reproduce. The evolutionary dynamics of the population can be described by restricting Equation 2 to the set of viable genotypes:
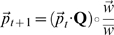
(3)where ‘

’ means entry-by-entry multiplication of the two vectors, 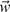 is a vector of the viabilities of each genotypic class (1 and 0 correspond to viable and inviable, respectively) and 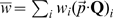 is the mean viability of the population.

**Figure 3:**
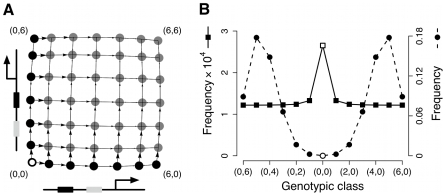
Condensed mutational networks for a promoter with 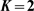 binding sites (both with length 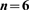). (A) Axes represent the Hamming distance of each binding site from the canonical sequence 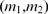. Each node represents a genotypic class. At each condensed genotypic class 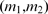 there are 
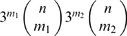
 genotypes. The magnitude of the probability of transition between two genotypic classes is denoted by the length of the arrowheads. In this example, the promoter regulates an essential gene such that at least one canonical binding site is required for activity (see Equation 4). The nodes in black define the viable portion of the condensed mutational network. The open circle denotes the redundant genotype. The nodes in gray represent inviable genotypes. (B) Shows the equilibrium distribution over the viable portion of the condensed mutational network. Squares (solid line, left axis) show the average frequencies of *each* genotype in a genotypic class; circles (dashed line, right axis) show the sum of the frequencies of *all* genotypes in a genotypic class. The redundant genotype (0,0) is shown in an open symbol, all other (nonredundant) genotypes are represented by closed symbols.

Equations 2 and 3 can be generalized for genotypes with any number of binding sites *K* (Figures 3A, S2 and S3). This amounts to considering that each possible binding site defines an axis in a *K*-dimensional space, and that each point along that axis is the Hamming distance between the site and the canonical binding sequence of the corresponding TF. In the next four sections we consider the 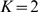 case in detail (see ‘Number of segregating binding sites’ for 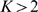).

### Full redundancy

We begin by considering one of the simplest situations that can be represented in our model: an essential gene regulated by a single constitutively expressed activator 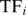. Gene function is required for viability. A binding site for this TF, 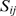, is functional only if it matches the canonical binding sequence exactly 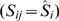:
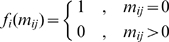
(4)A single functional binding site is both necessary and sufficient to sustain gene function; additional functional binding sites are *fully* redundant.

The condensed mutational network for the case of 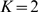 sites is shown in Figure 3A. The axes represent the Hamming distances of each binding site relative to the canonical sequence. The viable portion of the condensed mutational network comprises the genotypic classes that have at least one functional site, and corresponds to the left and bottom edges of the mutational network in Figure 3A. There is only one redundant genotypic class: that possessing two functional binding sites (open circle in Figure 3A).

Not all genotypes within the viable portion of the condensed mutational network have the same reproductive value, defined as the proportion of viable offspring they produce. The reproductive value of condensed genotypic class *i* is given by: 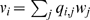. The redundant genotype (0,0) has a reproductive value of 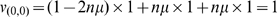 because both of its mutational neighbors, (0,1) and (1,0), are viable. All other viable genotypes (with one functional binding site) have reproductive value 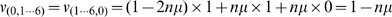 (i.e., half of its mutational neighbors are inviable).

When the population reaches mutation-selection equilibrium it is not evenly distributed over all viable genotypes. Rather, the redundant genotype is approximately 2-fold overrepresented in the population, relative to other (nonredundant) genotypes (Figure 3B, squares). This finding is consistent with the prediction [20] that more highly connected genotypes in a neutral network should be overrepresented at equilibrium relative to a uniform distribution. But although the redundant genotype is overrepresented at equilibrium, redundancy cannot evolve easily in this model. That is because the (noncondensed) set of viable genotypes contains a single redundant genotype, but 8,190 nonredundant ones, 83% of which include at least 4 mismatches in the nonfunctional binding site. Thus, at equilibrium, the redundant genotype constitutes a miniscule proportion of the population (0.012%). This pattern is visible in the sum of the frequencies of all genotypes in a viable condensed genotypic class (Figure 3B, circles).

The model outlined above can be considered *neutral* with respect to redundancy because redundant and nonredundant genotypes have the same viability [9], [20]. The interaction between viability selection and the structure of the mutational network in this model does create indirect selection for multiplicity [20], but it is too weak to maintain a substantial proportion of redundant genotypes in the population. The results in this section are consistent with those obtained by Gerland and Hwa using a similar model [4]. In the next three sections, we build on this model by introducing different mechanisms independently, one at a time, and investigating how they affect the evolution of redundant genotypes.

### Partial redundancy

**Figure 4 pcbi-1000848-g004:**
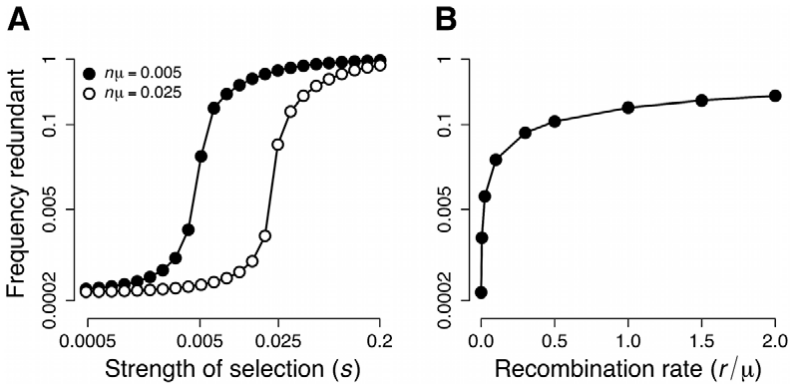
Effects of selection for multiplicity and recombination on the evolution of redundant *cis*-regulation. Each effect was added separately to the model shown in Figure 3. Values are the total frequencies of the redundant genotype (0,0) at equilibrium under different scenarios. (A) Direct selection for multiplicity (partial redundancy) under different mutation rates, 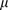. (B) Recombination rate between binding sites, 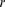. The equilibrium distribution is invariant for a given 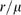, but it is reached more quickly for higher values of 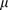.

*Partial* redundancy is thought to be more common than full redundancy [12], [15]. The presence of multiple binding sites might be advantageous if, for example, it changes the expression level of the target gene, or buffers expression against fluctuations in TF concentration [1], [3]–[5]. We model partial redundancy by setting the viabilities of redundant and nonredundant genotypes to 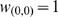 and 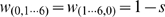, respectively. The equilibrium frequency of the redundant genotype increases with the strength of selection for redundancy (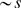; Figure 4A). The effect of selection on redundancy undergoes a phase transition around the point where selection becomes strong relative to the rate of mutation from redundant to nonredundant genotypes (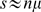): the response to selection is small for weaker selection, but it increases sharply for stronger selection.

### Recombination

We incorporate recombination into our full redundancy model by taking into account the probability that each genotype has resulted from recombination between each available pair of genotypes (see Protocol S1 for details). Recombination is only allowed *between* binding sites, not within them. Recombination changes the evolutionary dynamics because it allows long steps across the mutational network. A modest amount of recombination between sites (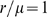) leads to the evolution of a high level of redundancy at mutation-recombination-selection equlibrium (Figure 4B). Lynch obtained similar results using a simpler model [9].

Our result can be understood by considering recombination between nonredundant genotypes containing different functional binding sites, that is, 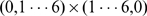 in Figure 3A. A recombination event between the sites produces two genotypes: one viable, with two functional binding sites (redundant), and another inviable, without any functional sites. In contrast, redundant genotypes always give rise to viable offspring, regardless of the kind of genotype they recombine with. This leads to strong selection against nonredundant genotypes. Stochastic simulations confirm that recombination can have a large effect on the evolution of redundancy even in finite populations (Figure S4).

### TF promiscuity

Many TFs are promiscuous, that is, they can bind to several different sequences (Figure 1). Our model allows us to explore the implications of different levels and kinds of TF promiscuity for the evolution of redundancy (Figure S2B; also, see next section). We consider two different ways of increasing the promiscuity of the TF described in the basic full redundancy model (see Equation 4).

**Figure 5:**
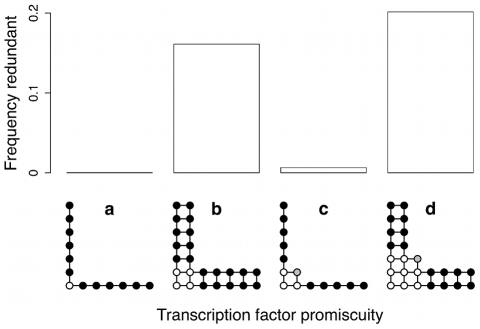
TF promiscuity promotes the evolution of redundancy. (a–d) Viable portions of the condensed mutational networks corresponding to different kinds of TFs. Circles represent viable genotypes. White circles denote redundant genotypes, with two functional binding sites; gray circles denote nonredundant genotypes with two binding site that are functional when acting together, but not in isolation; black circles denote nonredundant genotypes with one functional binding site. See Figure S1 for examples of *f* functions consistent with these condensed mutational networks. The bars show the equilibrium frequencies of redundant genotypes. The TF represented in (a) is the same as that shown in Figure 3 (non-promiscuous). (b) Represents an “all or nothing” promiscuous TF that allows binding sites with one mismatch without losing regulatory influence (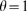 in Equation 5). (c) and (d) show examples of “graded” TF promiscuity. In (c) two binding sites with 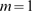 mismatch are functional when acting together, but not in isolation. In (d) two binding sites with 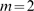 mismatches are functional when acting together, but not in isolation. All binding sites have length 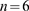.

We begin by considering the “all or nothing” case where 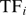 affects gene expression through a binding site 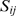 according to the following relationship:
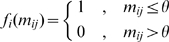
(5)The binding site is functional only if its sequence differs from the canonical sequence of 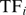 by no more than 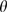 mismatches (Equation 4 is the special case for 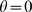). Thus, the greater the value of 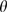, the more promiscuous the TF. Increasing 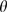 expands both the size of the viable portion of the condensed mutational network and the number of redundant states. Figure 5 shows the viable portions of the condensed mutational networks for a stringent (a: 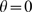) and a promiscuous TF (b: 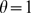). The promiscuous TF evolves an equilibrium frequency of redundant genotypes two orders of magnitude greater than the stringent one.

Generally, mismatches reduce the binding affinity and, therefore, the regulatory influence of a TF [1], [26], [29]. For example, in the *D. melanogaster eve* stripe 2 enhancer, the different *bcd* binding sites show different numbers of mismatches relative to the *bcd* consensus recognition sequence, inferred from *in vitro* binding assays [2], [30] (Figure 1). The deletion of binding sites with lower numbers of mismatches (B1 or B2, both with 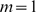) result in much more severe reductions in stripe 2 expression, when compared to deletions in sites with higher numbers of mismatches (B4 or B5, both with 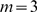; B3, with 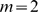) [18], [31]. In addition, when the high-*m* sites B3–B5 were mutated into consensus sites (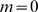), they restored expression of a defective promoter lacking the B1 binding site [18]. To incorporate this type of “graded” TF promiscuity in our model, we defined 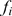 as a decreasing function of 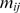 (instead of a step-function as in Equation 5). Figure 5 shows two examples (c, d) that imply that graded promiscuity can promote the evolution of redundant *cis*-regulation more strongly than the all or nothing kind. The reason for this is that graded TF promiscuity can lead to the appearance of nonredundant genotypes containing multiple binding sites capable of sustaining gene function together but not in isolation (gray, Figure 5). These results show that nonredundant multiplicity can evolve from redundant transitional forms.

### Number of segregating binding sites

Until now, our model has assumed that only alleles at 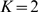 binding sites segregate within a population at a given time. This assumption may not be met in reality. Long promoters provide the opportunity for more sites to arise by chance in a population [10] (Figure S2). Other factors that are expected to influence the number of segregating binding site alleles include the length of the site (*n*, Figure S2A), the match between the GC-content of the promoter and that of the canonical binding sequence [10], the promiscuity of the TF (*m*, Figure S2B; see previous section), the mutation rate and the population size.

As the number of segregating binding sites in the full redundancy model increases, the dimensionality of the model and the number of possible redundant genotypes also increase (Figures S2C and S3). The increase in the number of available redundant genotypes results in an increase in the total equilibrium frequency of these genotypes (Figure S2D). But although the number of redundant genotypes grows roughly exponentially with the number of segregating binding site alleles, the equilibrium frequency of redundant genotypes increases linearly, suggesting that the number of segregating binding site alleles has only a modest effect on the evolution of redundancy. The situation changes when the expected binding site copy number in a sequence is 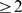 (e.g., points above the dashed line in Figures S2A and S2B). If that occurs, the maintenance of redundant *cis*-regulation does not require a selective explanation.

### Generalizations and caveats

All the results derived above for an individual transcriptional activator can be generalized to two scenarios. First, to combinations of *different* transcriptional activators following similar rules. This would allow us to model the evolution of *cis*-regulatory element *degeneracy* (the equivalent of redundancy for elements that are structurally different [14]). Second, to transcriptional repressors (considered individually or in combination), where the function of the target gene is defined by its *inactivity*. Selection for *decreased* gene expression is expected to influence the evolution of the copy number of the binding sites of transcriptional *repressors* in the same way that selection for *increased* gene expression affects the evolution of the copy number of the binding sites of transcriptional *activators*. A major challenge for future work is to consider the simultaneous evolution of sites for activators and repressors in the same promoter.

Our model includes many simplifying assumptions, such as that the positions within a binding site influence TF binding uniformly and additively, and that TFs act additively through multiple binding sites. Additivity among the positions of a binding site appears to be a reasonable approximation [29], [32] and (together with uniformity) serves as the basis for the widely used two-state model [4], [25]–[27]. The other assumptions are not particularly realistic: synergistic effects among binding sites are commonplace [33], [34] and many TFs are not uniformly promiscuous (Table S1). The extent to which changing the assumptions of our model would modify our conclusions is not clear at present, and remains a fundamental question for future modeling.

## Results

### *Cis*-regulatory element multiplicity in yeast

To evaluate the level of regulatory multiplicity in the yeast genome, we have scanned all intergenic sequences depleted of nucleosomes [35] upstream of a single protein-coding gene (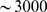 sequences, covering 8% of the genome) for 326 position weight matrix (PWM) models of 179 TFs from the literature [28], [36]–[38] (see Methods; Tables S1 and S2). In what follows, we analyse the 312 PWMs (corresponding to 176 TFs) predicted to have at least two binding sites in total. For simplicity, we refer to intergenic regions as promoters. On average, each promoter contained 0.08 binding sites of each PWM (standard deviation, 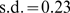).

**Figure 6:**
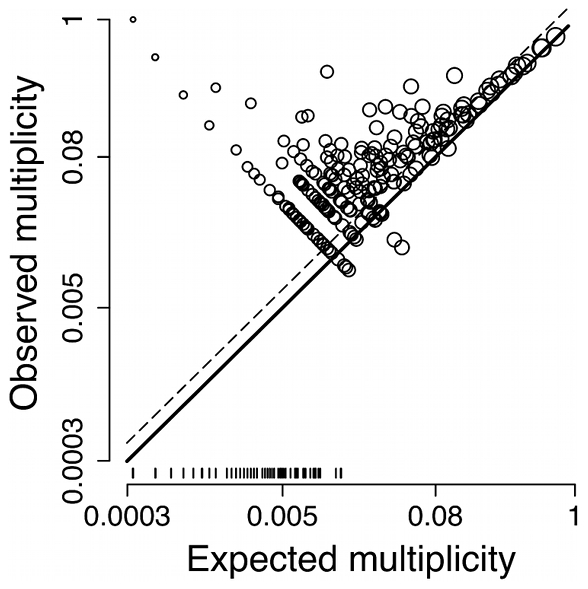
The yeast genome shows excess *cis*-regulatory element multiplicity. Observed multiplicity (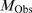) of the binding site distribution corresponding to each PWM against the multiplicity expected under a Poisson distribution (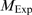) (data in Table S3). Axes are log-transformed. Values of *M* vary between 0 (all binding sites found in single copies in different intergenic regions) and 1 (all binding sites found in multiple copies). The area of the circles is proportional to the log of the total binding site count for the PWM. The bold line shows the expectation 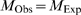; the dashed line shows the median excess multiplicity (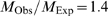). Vertical lines denote cases where 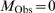.

We defined the amount of regulatory multiplicity (*M*) for a PWM as the proportion of promoters having at least one binding site that have two or more binding sites. On average, PWMs showed 7.1% multiplicity (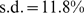; Table S3). The *M* measure of multiplicity is partly confounded with overall binding site copy number. To correct for this effect, we calculated the expected value of *M* for each PWM under the assumption that binding site copy number in a promoter region *i* of length 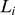 (nucleosome depleted) is Poisson distributed with expectation 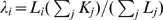, where 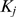 is the observed number of binding sites in promoter *j*. Figure 6 shows that the observed *cis*-regulatory element multiplicity was approximately 40% higher than that expected under the null expectation (paired Wilcoxon test of the hypothesis that 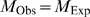: 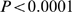).

### Evolutionary mechanisms

**Figure 7:**
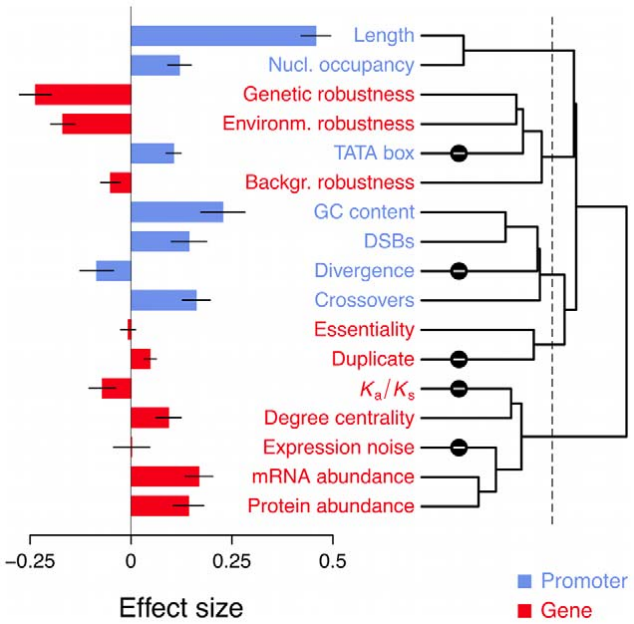
*Cis*-regulatory element multiplicity is associated with recombination and other genomic features. Relationship between regulatory multiplicity and different genomic features. Bars show the mean and 95% confidence intervals for the effect sizes of the difference between promoters with multiple and single binding sites [39]. The estimates were obtained from a random-effects meta-analytic model fitted using REML. Blue and red bars denote features of the promoter regions and of the genes downstream of these regions, respectively (see Methods for details). Positive values indicate that promoters showing multiple binding site multiplicity tend to show high values of the feature. Promoter length was log-transformed. The dendrogram summarizes the pattern of correlations between different features and was constructed by applying Ward's hierarchical clustering algorithm to a dissimilarity matrix composed of 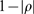, where 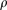 is Spearman's correlation coefficient (see Table S4). Negative signs in the branches leading to a certain feature (e.g., TATA box) indicate that that feature is negatively correlated (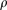) with other features belonging to a cluster defined by the dashed line.

How did the excess multiplicity shown in Figure 6 evolve? Our model and the literature suggest three possibilities [1], [3]–[5], [9]: 1) recombination, 2) direct selection for increased robustness in gene expression, or 3) direct selection for increased gene expression. Each hypothesis makes a different prediction about promoters displaying binding site multiplicity: they should experience 1) higher *recombination* rates, or be upstream of genes showing 2) more *robust* expression patterns, or 3) higher *expression* levels (for activators; the opposite is expected for repressors). To test these hypotheses, we looked for genome-wide associations between *cis*-regulatory element multiplicity and a range of features of the promoters and the genes downstream of those promoters. Consider a genomic property 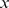 (e.g., promoter length). For each PWM, we calculated an effect size 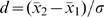, where 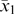 (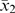) is the mean of 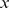 associated with promoters containing a single (multiple) binding site(s), and 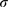 is an unbiased estimate of the pooled standard deviation [39] (the effect size for binary traits, such as gene essentiality, was estimated as the arcsine transformed risk difference based on 2
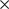
2 tables). We then combined the effect sizes and respective variances corresponding to different PWMs in a random-effects meta-analytic model. The results are summarized in Figure 7.

Promoters with higher numbers of crossovers [40] showed significantly higher levels of binding site multiplicity (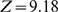, 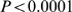; Figure 7), which is consistent with the *recombination* hypothesis. This association is explained in part by promoter length (Spearman's rank correlation coefficient: 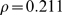, 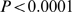). However, we believe that our data provide strong backing for the *recombination* hypothesis for three reasons. First, promoter length alone cannot explain the excess multiplicity illustrated in Figure 6 because it was considered in the calculation of 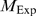. When the analysis was restricted to the subset of PWMs displaying excess multiplicity (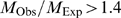), the effect size of crossover number was unchanged (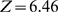, 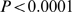). Second, the effect size of the residual crossover number from a Poisson log-linear regression model with log-transformed promoter length as an explanatory variable decreased (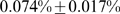), but remained statistically significant (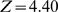, 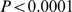). Third, a measure of frequency of meiotic double-strand breaks (DSBs) per bp [41] was also elevated in promoters showing *cis*-regulatory element multiplicity (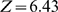, 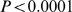; Figure 7).

Promoters showing binding site multiplicity tended to be upstream of genes showing low robustness in gene expression to various trans-perturbations [42] (all 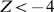, 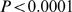, which contradicts the hypothesis that redundancy has evolved as a result of *selection for robustness* in gene expression. Although promoters with multiple binding sites were also more likely to contain a TATA box [43] (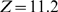, 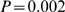), the results shown in Figure 7 did not change qualitatively when the analyses were repeated separately for genes with and without TATA boxes (not shown). Furthermore, multiplicity was not associated with protein expression noise [44] (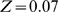, 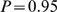. Genes downstream of promoters with multiple binding sites tended to have higher expression levels (both protein and mRNA: 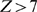, 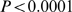), which is consistent with the *selection for expression* hypothesis for activators, but not repressors.

*Cis*-regulatory element multiplicity was associated with several correlates of gene functionality (Figure 7). Promoters containing multiple sites tended to evolve more slowly [45] (divergence: 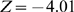, 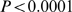), and the genes downstream of these promoters tended to show higher levels of selective constraint [45] (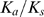: 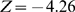, 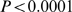) and to be involved in interactions with a greater number of other genes [46] (degree centrality: 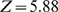, 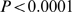). Genes with duplicates elsewhere in the genome were more likely to show binding site multiplicity [47] (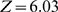, 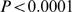). Several gene ontology terms were significantly enriched in genes downstream of promoters containing multiple sites, including: plasma membrane, transporter activity, transcription regulator activity, DNA binding and transport (Table S5).

## Discussion

### Partial redundancy

Our mathematical model suggests that selection for multiple binding sites, that is, partial redundancy, can influence the evolution of *cis*-regulatory element multiplicity, provided that the redundant genotype has a selective advantage 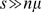. Mutation rates per base pair in DNA-based organisms are of the order of 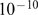 [48]. Therefore, weak selection can play a major role in the evolution of *cis*-regulatory element multiplicity, provided that the effective population size is also large enough (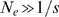) to render genetic drift negligible [4], [49].

We found a positive association in yeast between the presence of multiple binding sites for a TF and expression level of the downstream gene. This association is unlikely to have evolved neutrally or as a correlated response to the increases in multiplicity generated by recombination (see below) because gene expression patterns are under intense stabilizing selection [50], [51] and increases in gene expression are energetically costly [52]; also, the effect sizes of crossover number are not significantly correlated with those of either mRNA or protein abundance (both 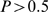). Rather, the association between multiplicity and gene expression is consistent with an adaptive origin of *cis*-regulatory element multiplicity. Increasing the number of binding sites for transcriptional activators (inhibitors) in a promoter typically increases (decreases) gene expression [34], [53]. Since transcriptional activators are thought to be 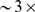 more common than repressors in yeast, and many TFs can perform either role [54], selection for different levels of expression of certain genes in certain environments could, over time, generate a positive association between *cis*-regulatory element multiplicity and expression level (provided that there is no strong overall bias towards selection for reduced expression). This adaptive scenario for the evolution of binding site multiplicity is consistent with the observation that functional TF binding sites have been frequently gained or lost in a lineage-specific manner among three closely related species of yeast [55], [56].

Our finding that *cis*-regulatory element multiplicity was associated with *lower* robustness in gene expression to various trans-perturbations contradicts the hypothesis that redundant genotypes benefit from being more robust [1], [5]. An earlier study reported a positive association between the number of binding sites for *any* TF—a possible correlate of *both* redundancy and degeneracy [14]—and variation in gene expression in yeast using different data from ours [57]. Our evidence is, of course, correlative: a more direct test would be to compare the robustness in expression of genes downstream of promoters containing multiple binding sites with that of the same genes with various combinations of sites mutated or deleted. Nevertheless, the observed relationships between multiplicity and robustness are also consistent with selection for changes in expression level. If the main consequence of gaining binding sites is to increase the effect of a TF on gene expression (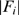 in Equation 1), then changes in the levels of TFs, such as those caused by viable knockout mutations [42], are expected to lead to greater variance in these effects in promoters containing multiple sites, compared to promoters containing a single binding site.

### Full redundancy

In addition, our model highlighted three candidate nonadaptive mechanisms for the evolution of *cis*-regulatory element multiplicity through fully redundant transitional forms. The first is the neutral evolution of multiple binding sites in long promoters. Such “trivial” redundancy is expected to occur in a long promoter if functional binding sites can occur over a large proportion of its length. Although the latter condition is difficult to evaluate in real organisms, intergenic regions longer than 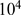-bp are common in several mammals, including humans. Therefore, many mammalian promoters may be trivially redundant [9], [10]. This could explain the observation that approximately a third of human functional TF binding sites are not functional in rodents [58]. However, we do not expect that trivial redundancy played a dominant role in the evolution of multiplicity in organisms with relatively shorter promoters and larger populations, such as yeast.

The second mechanism is recombination. Based on our model we predict that recombination between binding sites on the order of 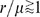 will promote the evolution of *cis*-regulatory element redundancy. In yeast, a pair of sites 100-bp apart is expected to experience 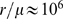 [48], [59]; if yeast only undergo sexual reproduction once every 1,000 asexual generations [60] we estimate 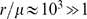, suggesting that this process has operated in yeast. We found a positive association between the presence of multiple binding sites for a TF and recombination rate in yeast. Estimates based on polymorphism data from 10 species of plants and animals [49] give 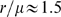 bp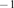, indicating that recombination is likely to be a powerful force in the evolution of *cis*-regulatory element multiplicity in other eukaryotes with relatively large populations. Our findings are in agreement with recent work showing that recombination selects for “mixable” genotypes [61], which leads to the evolution of higher mutational robustness [8], [62]–[64]. Our model predicts that redundant genotypes are robust to mutations in the binding sites, but this kind of mutational robustness does not imply robustness in the expression pattern of the downstream gene to trans-perturbations. In fact we found that *cis*-regulatory element multiplicity was associated with *reduced* robustness to perturbations in trans (see previous section).

The third nonadaptive mechanism indicated by our model is that increases in TF promiscuity promote the evolution of *cis*-regulatory element multiplicity. We could not test this prediction directly with our yeast data because we made an implicit assumption about the level of TF promiscuity when we scanned for binding sites. However, Bilu and Barkai [57], using a different data set from ours, reported that binding sites tended to be “fuzzier” (i.e., have lower PWM scores) when they appeared in promoter regions containing other binding sites for any TF. This observation is consistent with the prediction that graded TF promiscuity allows the existence of viable genotypes containing multiple binding sites, where each binding site is fuzzier than those found in viable genotypes containing fewer binding sites. Graded TF promiscuity is believed to be common [1], [26], [29], suggesting that multiplicity will often evolve through transitional forms showing redundant *cis*-regulation that then degenerate into nonredundant forms. If this evolutionary scenario is common, then lack of redundancy in extant genotypes containing multiple binding sites will be a poor indicator of whether or not its ancestral genotypes were redundant.

### Conclusion

Our results suggest that redundant transitional forms can, indeed, play an important role in the evolution of *cis*-regulatory element multiplicity. Many aspects of the biology of an organism affect the evolution of redundancy and multiplicity: both adaptive and nonadaptive processes, both changes in *cis* to binding sites and in trans to the TFs that interact with them, both the functional setting of the promoter and the population genetic context of the individuals carrying them. Thus, understanding how gene networks evolve will require going beyond mere plausibility arguments into rigorous testing of specific mechanisms [4], [9]. We believe that the approach developed here provides a valuable framework to advance this research program.

## Methods

### Model analysis

The results reported in Figures 3–5 and were based on a deterministic version of the model (i.e., assuming infinite population size). The frequencies of different genotypic classes at mutation-selection or mutation-recombination-selection equilibrium were calculated by iterating populations for as long as necessary for genotypic class frequencies not to change by more than 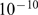 from one generation to the next.

### TF binding site models

We used 326 PWMs summarizing the binding specificities of 179 putative yeast TFs reported in four studies [28], [36]–[38] (Table S1; see Protocol S1 for more details). Sequences scoring 95% or higher of the highest possible score for a given PWM were considered putative binding sites (on average, this allowed 1.23 mismatches, s.d. = 1.51). Each intergenic sequence was scanned with a PWM and its reverse complement and the number of matches were counted (simultaneous hits on exactly the same sequence and its reverse complement 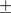
1 nucleotide were counted as a single hit; otherwise, binding sites overlapping over 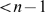 nucleotides were counted separately).

Other studies have attempted to distinguish between real and “impostor” binding sites by taking into account additional information, such as the degree of conservation of putative sites [36], [37]. We did not follow this approach because promoter sequence divergence is significantly correlated with many of the genomic features shown in Figure 7 (Table S4; see next section).

### Genomic features

We calculated the following quantities for each intergenic region: 1) sequence length (including regions occupied by nucleosomes); 2) proportion of sequence occupied by nucleosomes [35]; 3) whether it contains a TATA box [43]. 4) GC content of the sequence; 5) a measure of the frequency of meiotic DSBs [41]; 6) proportion of nucleotides that differ between *S. cerevisiae* and *S. paradoxus* [45]. 7) number of crossover events [40]. We also calculated the following quantities for the gene downstream of these promoters: 1) three measures of robustness to trans-perturbations [42], derived from measurements of the variance in levels of gene expression (corrected for mean) across 167 viable knockout mutations (genetic), 30 wild isolates (genetic background), and 35 environments (environmental robustness); 2) essentiality, whether a homozygous knock-out of the gene was lethal [65], [66]; 3) whether the gene has a duplicate elsewhere in the genome [47]; 4) 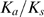, the ratio between the rates of nonsynonymous and synonymous site substitution based on the comparison between *S. cerevisiae* and *S. paradoxus* [45]; 5) degree centrality, the total number of interactions with other genes [46]; 6) protein expression noise [44]; 7) mRNA and 10) protein abundance [67], [68]. See Protocol S1 for more details.

### Software

The model was analysed using Mathematica 6 (http://www.wolfram.com/mathematica/). Sequence and statistical analyses were done using R 2.9.0 (http://www.r-project.org/) and Bioconductor 2.4 (http://www.bioconductor.org/).

## Supporting Information

Protocol S1 Supplementary Methods. Sections: modeling recombination; yeast data; software.
(0.09 MB PDF)

Figure S1 Examples of *f* functions consistent with the viable portions of the condensed mutational networks in Figure 5. The dashed line indicates the threshold for driving gene expression.
(0.06 MB PDF)

Figure S2 Redundancy is more likely to evolve if there are more segregating binding site alleles. (A) Expected number of exact matches to canonical binding sequences of different lengths (*n*) in promoters of different lengths (*L*). (B) Expected number of matches to an 8-bp canonical binding sequence allowing for different numbers of mismatches (*m*) in promoters of different *L*. The value of *m* models different levels of TF promiscuity. In (A) and (B) values are means and 95% confidence intervals of 10 independent sets of 104 random sequences with the same average GC content as yeast intergenic regions (except for *n*  =  8, *m*  =  0 and *L*≤200, where 60 sets of sequences were used). Dashed lines mark an expected number of 2 binding sites. (C) Number of redundant genotypes and (D) total equilibrium frequency of redundant genotypes for different numbers of segregating binding sites (*K*). For *K*  =  2, the model is that shown in Figure 2. See Figure S3 for *K*  =  3.
(0.09 MB PDF)

Figure S3 Condensed mutational networks for a promoter with *K*  =  3 binding sites (all with length *n*  =  6). (A) Diagram of gene with three binding sites. (B) Condensed mutational network. Axes represent the numbers of mismatches of each binding site relative to the canonical sequence. Each node represents a genotypic class. As in Figure 2, the promoter regulates an essential gene such that at least one canonical binding site is required for activity. The nodes shown in black define the viable portion of the condensed mutational network. The nodes in gray represent inviable genotypes. (C) Shows only the viable portion of the condensed mutational network. The genotypes highlighted in gray are redundant.
(0.17 MB PDF)

Figure S4 Stochastic simulations of the effect of recombination. Populations of different sizes (*N*) are initialized at mutation-selection equilibrium. (A) *r/μ*  =  0.1, (B) *r/μ*  =  1, and (C) *r/μ*  =  10. In all cases, we used *μ*  =  0.1, an unrealistically high value. Values are medians of the frequencies of redundant genotypes for 500 replicate populations. In populations of both sizes redundancy evolves quickly, but is then lost by drift. Dotted lines show the deterministic expectation (see Figure 3B).
(0.08 MB PDF)

Table S1 Binding site PWM models used in our study. Values are the length (8.7 ± 2.6 bp, mean ± standard deviation), GC content (0.53 ± 0.19) and mean information content (*I*) per position (1.33 ± 0.29) of each PWM (after processing as described in Protocol S1). The value of *I* can vary between 0 and 2, and is a measure of the energy contribution of a position to TF binding. Each PWM is summarized by its canonical sequence: “.” indicates a position with *I*  =  0; “[ / ]” indicates bases with the same weight at a given position. Letters in parentheses after TF names indicate the study from which we took the PWM data: B, Badis et al. (2008); H, Harbison et al. (2004); M, MacIsaac et al. (2006); Zhu et al. (2009).
(0.06 MB PDF)

Table S2 Binding site PWM models not considered in our study. These PWMs were excluded because they were almost identical to the PWMs listed in the ‘Equivalent’ column, shown in Table S1. Letters in parentheses after TF names indicate the study from which we took the PWM data: B, Badis et al. (2008); H, Harbison et al. (2004); M, MacIsaac et al. (2006); Zhu et al. (2009).
(0.04 MB PDF)

Table S3 Data used to construct Figure 6. The data are sorted by decreasing *M*_Obs_/*M*_Exp_. The second and third columns show the Total number of binding sites revealed in a scan across the number of promoter regions in the ‘Prom’ column (the numbers vary because the minimum promoter length considered in each scan is twice the length of the PWM). Letters in parentheses after TF names indicate the study from which we took the PWM data: B, Badis et al. (2008); H, Harbison et al. (2004); M, MacIsaac et al. (2006); Zhu et al. (2009).
(0.05 MB PDF)

Table S4 Matrix of correlations between genomic features. Values are Spearman's rank correlation coefficients (*ρ*). Data used for the cluster analysis in Figure 7.
(0.04 MB PDF)

Table S5 Association between multiplicity and GO slim terms from each domain. Significance levels after correction for multiple comparisons using the Holm method: *, *P*<0.01; **, *P*<0.001; ***, *P*<0.0001.
(0.06 MB PDF)
