# Supplementary material for: Redundancy and the Evolution of Cis-Regulatory Element Multiplicity
Source: PLoS Comput Biol. 2010 Jul 8;6(7):e1000848. doi: 10.1371/journal.pcbi.1000848 (PMC2900288; doi:10.1371/journal.pcbi.1000848)
Supplement: Protocol S1 — Supplementary Methods. Sections: modeling recombination; yeast data; software. (0.09 MB PDF) [file pcbi.1000848.s001.pdf]

Protocol S1

Modeling recombination

We begin by constructing mating matrices $\mathbf{R}_k$ for each genotypic class k. The component $\mathbf{R}_{k;ij}$ is the probability that a mating between individuals in genotypic classes i and j will give rise to offspring belonging to class k. We weight these components by the probability that individuals in genotypic classes i and j encounter each other. Formally, this is included in the model by introducing a new operator "Rec" for recombination:

$$\mathrm{Rec}(\vec{p}) = \vec{p} \cdot \vec{\mathbf{R}} \cdot \vec{p}^{\,T} = \left(\vec{p} \cdot \mathbf{R}_0 \cdot \vec{p}^{\,T}, \cdots, \vec{p} \cdot \mathbf{R}_k \cdot \vec{p}^{\,T}\right)$$

We assume that the recombination rate within binding sites is zero. For example, considering the two binding site case, the probability that a mating between genotypic classes $(3,0)$ and $(0,5)$ produces the genotype $(0,0)$ is $\mathbf{R}_{(0,0);(3,0),(0,5)} = r/2$, where r is the recombination rate between binding sites. Different assumptions about how recombination operates can be represented by appropriate mating matrices. The evolutionary dynamics of an infinite sexual population is then described by:

$$\vec{p}_{t+1} = \mathrm{Rec}\left(\vec{p}_t \cdot \mathbf{Q}\right) \circ \frac{\vec{w}}{\overline{w}}$$

Yeast data

TF binding site models

We used 428 position weight matrices (PWMs) summarizing the binding specificities of 190 putative yeast TFs reported in four studies: two analyses of a single genome-wide chromatin immunoprecipitation data set [1, 2] and two independent protein binding microarray studies [3, 4] (Tables S1 and S2). Each PWM was simplified in two steps:

1. Sequential deletion of all terminal (both 5' and 3') positions with a total information content under 0.125.

2. Deletion of all but the 8 positions with the highest information content and intervening positions

We then compared all PWMs of the same length to each other by calculating the following distance:

$$d = \frac{\sum_{j=1}^{n} \sum_{i=1}^{4} (p_{ij} - p'_{ij})^2}{n}$$

where, p_{ij} is the frequency of nucleotide i at position j of the first PWM, and p'_{ij} is the corresponding position in the second PWM. The distance between the first PWM and the reverse complement of the

second PWM was also calculated and the smallest value of d was used. Any pairs of PWMs showing $d < 0.05$ were collapsed. Pairs of PWMs with equivalent consensus sequences at 100% or 95% of the maximum PWM score were also collapsed (Table S2). The final PWM data set consisted of 326 PWMs for 179 TFs (Table S1).

Mismatches

Scanning PWMs at 95% stringency means that certain low information positions allow mismatches. The maximum number of mismatches allowed by a PWM decreased with the mean information content per position (Spearman's $\rho = -0.682$, $P < 0.0001$) and increased with the length of the binding site ($\rho = 0.592$, $P < 0.0001$). Length and information content per position were, in turn, strongly negatively correlated with each other ($\rho = -0.504$, $P < 0.0001$).

Intergenic regions

We used the following sequence data: http://downloads.yeastgenome.org/sequence/genomic_sequence/intergenic/archive/NotFeature.20090220.fasta.gz.

Genomic features

We calculated the following quantities for each intergenic region:

1. Sequence length.
2. Proportion of sequence occupied by nucleosomes [5].
3. Whether the promoter contains a TATA box [6].
4. GC content. Positively correlated with recombinational activity [7] and nucleosome occupancy [5].
5. A measure of the frequency of meiotic double-strand breaks (DSBs) of a mutant (*dmc1*Δ) defective in DSB-repair obtained using microarray hybridization [8]; we used the mean of log-transformed unsmoothed average ratios of background-normalized fluorescence.
6. Proportion of nucleotides that differ between *S. cerevisiae* and *S. paradoxus* [9]. Based on the original alignments (http://www.broadinstitute.org/annotation/fungi/comp_yeasts/downloads.html).
7. Total number of crossover events identified from examining all four products of 56 yeast meioses [10].

We also calculated the following quantities for each gene downstream of these promoters:

1. Three measures of robustness to trans-perturbations [11], derived from measurements of the variance (corrected for the mean) in levels of gene expression across: 167 viable knockout mutations (genetic), 30 wild isolates (genetic background), and 35 environments (environmental robustness). The robustness metrics are inversely related to the variance in gene expression.

2. Essentiality—whether a homozygous knock-out of the gene is lethal). We merged the lists of essential genes in http://www-sequence.stanford.edu/group/yeast_deletion_project/Essential_ORFs.txt [12, 13] and the Comprehensive Yeast Genome Database (CYGD) [14]. We merged the lists of nonessential genes in CYGD and three other studies [15–17]. Overlaps between the resulting lists of essential and nonessential genes were reclassified as nonessential.

3. Whether the gene has a duplicate elsewhere in the genome [18].

4. The ratio between the rates of nonsynonymous and synonymous site substitution K_a/K_s based on the comparison between *S. cerevisiae* and *S. paradoxus* [9] (ftp://ftp-genome.wi.mit.edu/pub/annotation/fungi/comp_yeasts/S4.MutationCounts/b.KaKs_details.xls).

5. Degree centrality—total number of interactions between that gene and other genes, including transcription regulatory and protein-protein interactions [19].

6. Protein expression noise, defined as the average of the log-transformed coefficient of variation in protein expression in two environments [20]; inversely correlated with protein and mRNA abundance [20] (Fig. 5B).

7. mRNA abundance [21].

8. Protein abundance [22]. Estimates of mean protein abundance in this study are strongly correlated with those in [22].

Software

PWM scans were done using the Bioconductor package ‘Biostrings’. Effect size estimates and meta-analyses were done using the ‘metafor’ package in R [23]. Cluster analyses were done using the ‘cluster’ package in R.

References

1. Harbison CT, Gordon DB, Lee TI, Rinaldi NJ, Macisaac KD, et al. (2004) Transcriptional regulatory code of a eukaryotic genome. Nature 431: 99-104.

2. MacIsaac KD, Wang T, Gordon DB, Gifford DK, Stormo GD, et al. (2006) An improved map of conserved regulatory sites for *Saccharomyces cerevisiae*. BMC Bioinformatics 7: 113.

3. Badis G, Chan ET, van Bakel H, Pena-Castillo L, Tillo D, et al. (2008) A library of yeast transcription factor motifs reveals a widespread function for Rsc3 in targeting nucleosome exclusion at promoters. Mol Cell 32: 878–887.

4. Zhu C, Byers KJRP, McCord RP, Shi Z, Berger MF, et al. (2009) High-resolution DNA-binding specificity analysis of yeast transcription factors. Genome Res 19: 556–566.

5. Lee W, Tillo D, Bray N, Morse RH, Davis RW, et al. (2007) A high-resolution atlas of nucleosome occupancy in yeast. Nat Genet 39: 1235–1244.

6. Basehoar AD, Zanton SJ, Pugh BF (2004) Identification and distinct regulation of yeast TATA box-containing genes. Cell 116: 699–709.

7. Mieczkowski PA, Dominska M, Buck MJ, Gerton JL, Lieb JD, et al. (2006) Global analysis of the relationship between the binding of the Bas1p transcription factor and meiosis-specific double-strand DNA breaks in *Saccharomyces cerevisiae*. Mol Cell Biol 26: 1014–1027.

8. Buhler C, Borde V, Lichten M (2007) Mapping meiotic single-strand DNA reveals a new landscape of DNA double-strand breaks in *Saccharomyces cerevisiae*. PLoS Biol 5: e324.

9. Kellis M, Patterson N, Endrizzi M, Birren B, Lander ES (2003) Sequencing and comparison of yeast species to identify genes and regulatory elements. Nature 423: 241–254.

10. Mancera E, Bourgon R, Brozzi A, Huber W, Steinmetz LM (2008) High-resolution mapping of meiotic crossovers and non-crossovers in yeast. Nature 454: 479–485.

11. Proulx SR, Nuzhdin S, Promislow DEL (2007) Direct selection on genetic robustness revealed in the yeast transcriptome. PLoS ONE 2: e911.

12. Giaever G, Chu A, Ni L, Connelly C, Riles L, et al. (2002) Functional profiling of the *Saccharomyces cerevisiae* genome. Nature 418: 387–391.

13. Winzeler E, Shoemaker D, Astromoff A, Liang H, Anderson K, et al. (1999) Functional characterization of the *S. cerevisiae* genome by gene deletion and parallel analysis. Science 285: 901.

14. Güldener U, Münsterkötter M, Kastenmüller G, Strack N, van Helden J, et al. (2005) CYGD: the Comprehensive Yeast Genome Database. Nucl Acids Res 33: D364.

15. Levy S, Siegal M (2008) Network hubs buffer environmental variation in *Saccharomyces cerevisiae*. PLoS Biol 6: e264.

16. Deutschbauer AM, Jaramillo DF, Proctor M, Kumm J, Hillenmeyer ME, et al. (2005) Mechanisms of haploinsufficiency revealed by genome-wide profiling in yeast. Genetics 169: 1915–1925.

17. Ohya Y, Sese J, Yukawa M, Sano F, Nakatani Y, et al. (2005) High-dimensional and large-scale phenotyping of yeast mutants. Proc Natl Acad Sci U S A 102: 19015–19020.

18. Kellis M, Birren BW, Lander ES (2004) Proof and evolutionary analysis of ancient genome duplication in the yeast *Saccharomyces cerevisiae*. Nature 428: 617-24.

19. Lee I, Li Z, Marcotte E (2007) An improved, bias-reduced probabilistic functional gene network of baker's yeast, *Saccharomyces cerevisiae*. PLoS ONE 2: e988.

20. Newman JRS, Ghaemmaghami S, Ihmels J, Breslow DK, Noble M, et al. (2006) Single-cell proteomic analysis of *S. cerevisiae* reveals the architecture of biological noise. Nature 441: 840–846.

21. Holstege FCP, Jennings EG, Wyrick JJ, Lee TI, Hengartner CJ, et al. (1998) Dissecting the regulatory circuitry of a eukaryotic genome. Cell 95: 717–728.

22. Ghaemmaghami S, Huh W, Bower K, Howson RW, Belle A, et al. (2003) Global analysis of protein expression in yeast. Nature 425: 737–741.

23. Ihaka R, Gentleman R (1996) R: a language for data analysis and graphics. J Comp Graph Stat 5: 299-314.
