# Supplementary figures and images for: Redundancy and the Evolution of Cis-Regulatory Element Multiplicity
Source: PLoS Comput Biol. 2010 Jul 8;6(7):e1000848. doi: 10.1371/journal.pcbi.1000848 (PMC2900288; doi:10.1371/journal.pcbi.1000848)

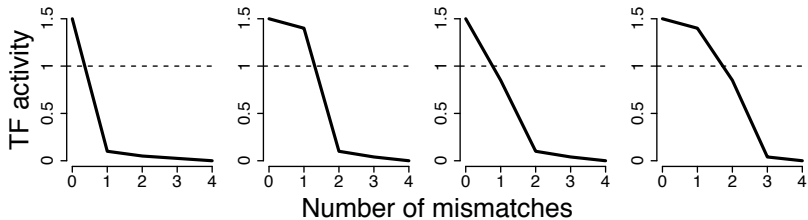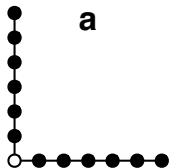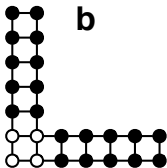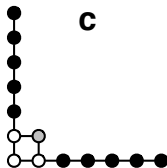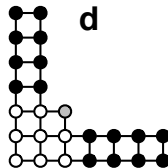

Supplement: Figure S1 — Examples of f functions consistent with the viable portions of the condensed mutational networks in Figure 5. The dashed line indicates the threshold for driving gene expression. (0.06 MB PDF) [file pcbi.1000848.s002.pdf]

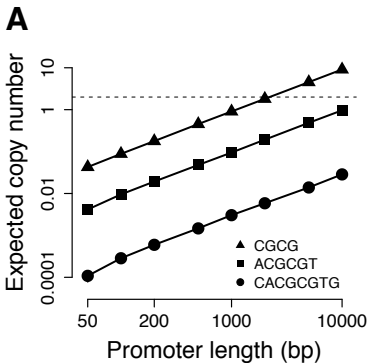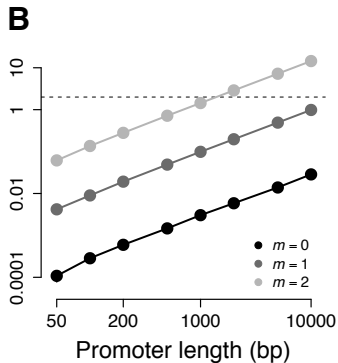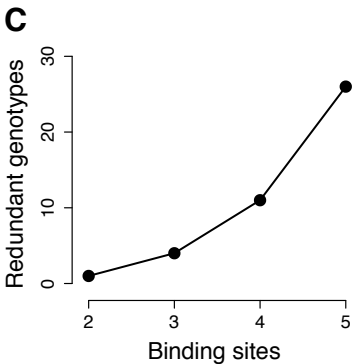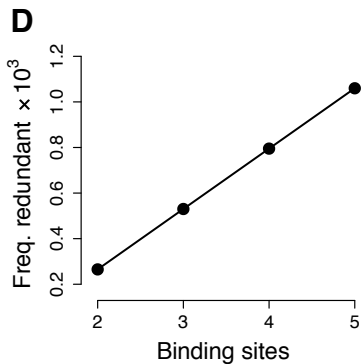

Supplement: Figure S2 — Redundancy is more likely to evolve if there are more segregating binding site alleles. (A) Expected number of exact matches to canonical binding sequences of different lengths (n) in promoters of different lengths (L). (B) Expected number of matches to an 8-bp canonical binding sequence allowing for different numbers of mismatches (m) in promoters of different L. The value of m models different levels of TF promiscuity. In (A) and (B) values are means and 95% confidence intervals of 10 independent sets of 104 random sequences with the same average GC content as yeast intergenic regions (except for n = 8, m = 0 and L≤200, where 60 sets of sequences were used). Dashed lines mark an expected number of 2 binding sites. (C) Number of redundant genotypes and (D) total equilibrium frequency of redundant genotypes for different numbers of segregating binding sites (K). For K = 2, the model is that shown in Figure 2. See Figure S3 for K = 3. (0.09 MB PDF) [file pcbi.1000848.s003.pdf]

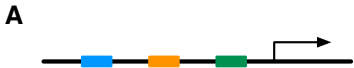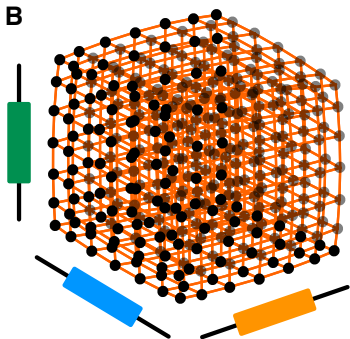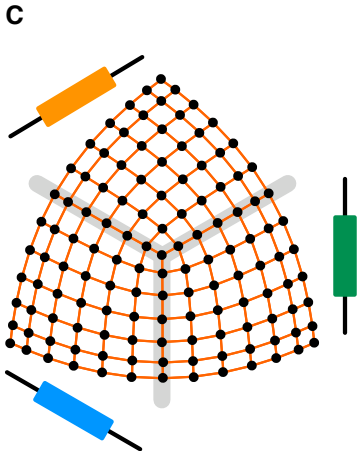

Supplement: Figure S3 — Condensed mutational networks for a promoter with K = 3 binding sites (all with length n = 6). (A) Diagram of gene with three binding sites. (B) Condensed mutational network. Axes represent the numbers of mismatches of each binding site relative to the canonical sequence. Each node represents a genotypic class. As in Figure 2, the promoter regulates an essential gene such that at least one canonical binding site is required for activity. The nodes shown in black define the viable portion of the condensed mutational network. The nodes in gray represent inviable genotypes. (C) Shows only the viable portion of the condensed mutational network. The genotypes highlighted in gray are redundant. (0.17 MB PDF) [file pcbi.1000848.s004.pdf]

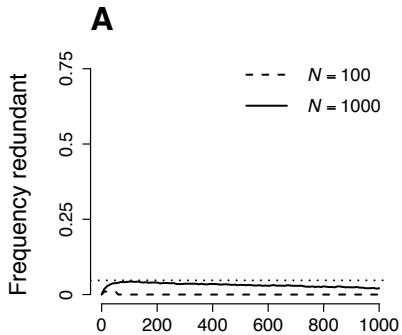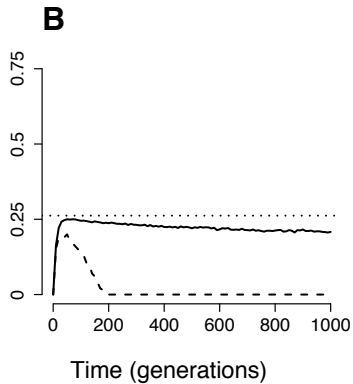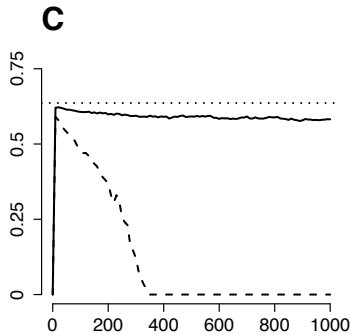

Supplement: Figure S4 — Stochastic simulations of the effect of recombination. Populations of different sizes (N) are initialized at mutation-selection equilibrium. (A) r/μ = 0.1, (B) r/μ = 1, and (C) r/μ = 10. In all cases, we used μ = 0.1, an unrealistically high value. Values are medians of the frequencies of redundant genotypes for 500 replicate populations. In populations of both sizes redundancy evolves quickly, but is then lost by drift. Dotted lines show the deterministic expectation (see Figure 3B). (0.08 MB PDF) [file pcbi.1000848.s005.pdf]
