# Supplementary material for: Redundancy and the Evolution of Cis-Regulatory Element Multiplicity
Source: PLoS Comput Biol. 2010 Jul 8;6(7):e1000848. doi: 10.1371/journal.pcbi.1000848 (PMC2900288; doi:10.1371/journal.pcbi.1000848)
Supplement: Table S1 — Binding site PWM models used in our study. Values are the length (8.7 ± 2.6 bp, mean ± standard deviation), GC content (0.53 ± 0.19) and mean information content (I) per position (1.33 ± 0.29) of each PWM (after processing as described in Protocol S1). The value of I can vary between 0 and 2, and is a measure of the energy contribution of a position to TF binding. Each PWM is summarized by its canonical sequence: “.” indicates a position with I = 0; “[ / ]” indicates bases with the same weight at a given position. Letters in parentheses after TF names indicate the study from which we took the PWM data: B, Badis et al. (2008); H, Harbison et al. (2004); M, MacIsaac et al. (2006); Zhu et al. (2009). (0.06 MB PDF) [file pcbi.1000848.s006.pdf]

Table S1. Binding site PWM models used in our study. Values are the length (8.7±2.6 bp, mean±s.d.), GC content (0.53±0.19) and mean information content (I) per position (1.33±0.29) of each PWM (after processing as described in Protocol S1). The value of I can vary between 0 and 2, and is a measure of the energy contribution of a position to TF binding. Each PWM is summarized by its canonical sequence: "." indicates a position with $I = 0$; "[/]" indicates bases with the same weight at a given position. Letters in parentheses after TF names indicate the study from which we took the PWM data: B, Badis *et al.* (2008); H, Harbison *et al.* (2004); M, MacIsaac *et al.* (2006); Zhu *et al.* (2009).

TF (Study)	Length	GC content	Information	Canonical sequence
ABF1 (B)	14	0.4965	0.810	TCGTATAAAGTGAT
ABF1 (H)	12	0.4035	0.745	TCACTATATACG
ABF1 (M)	13	0.4878	1.016	ATCACTATGCACG
ABF2 (B)	6	0.3571	1.772	TCTAGA
ACE2 (B)	7	0.7096	1.362	ACCAGCA
ACE2 (M)	8	0.6029	0.876	AACCAGCA
ADR1 (H)	5	0.7856	1.366	GG[A/G]G[G/T]
AFT1 (Z)	9	0.5536	1.295	ATTGCACCC
AFT2 (B)	8	0.7140	1.437	CACACCCC
AFT2 (H)	6	0.7342	1.085	GGGTGC
AFT2 (M)	7	0.7480	1.217	GGCACCC
ARG80 (M)	6	0.5650	1.518	AGACGC
ARG81 (M)	8	0.4725	1.370	GTGACTCA
ARO80 (M)	22	0.6293	1.417	CGAGGGTAGCCGCGAGTTGCCG
ARO80 (Z)	8	0.5990	1.202	CTCGGCAA
ARR1 (H)	7	0.1601	1.719	TTACTAA
ARR1 (M)	8	0.2780	1.015	ACTTGAAT
ASG1 (B)	6	0.6713	1.479	CCGGAA
ASG1 (Z)	15	0.7371	0.851	CCGGCCGAGTTCCGG
ASH1 (H)	6	0.4206	1.573	[C/T]TGACT
ASH1 (M)	10	0.6674	1.025	CCGGATCGGG
AZF1 (B)	7	0.2411	1.520	AAAAGAA
AZF1 (H)	15	0.2743	0.969	TTTTTCTTTTTCTGT
AZF1 (M)	18	0.4340	0.810	TTTTTCTTTTCCTGTGTC
BAS1 (H)	6	0.4970	1.741	TGACTC
BAS1 (M)	7	0.5390	1.375	TGACTCG

Continued on next page

TF (Study)	Length	GC content	Information	Canonical sequence
BAS1 (Z)	9	0.6012	1.394	CCAGAGTCA
CAD1 (H)	9	0.2965	1.328	TTAGTAAGC
CAD1 (M)	10	0.3259	1.161	GCTTACTAAT
CAT8 (B)	5	0.8647	1.706	CCGGA
CBF1 (B)	8	0.5972	1.565	GCACGTGA
CBF1 (M)	8	0.6239	1.627	GTCACGTG
CBF1 (Z)	10	0.6033	1.112	GTCACGTGAC
CEP3 (B)	7	0.5263	1.652	CTCGGAA
CEP3 (Z)	8	0.4902	1.207	TTCGGAAA
CHA4 (B)	8	0.7500	1.399	GGCGGAGA
CHA4 (M)	8	0.5897	1.201	GCGATGAG
CHA4 (Z)	9	0.6319	1.199	ATCTCCGCC
CIN5 (H)	8	0.2178	1.152	TTACGTAA
CIN5 (M)	9	0.3193	0.978	CTTACGTAA
CRZ1 (B)	9	0.6821	0.917	CTAAGCCAC
CRZ1 (M)	7	0.7066	1.611	GAGGCTG
CST6 (B)	8	0.4605	1.419	ATGACGTA
CST6 (M)	6	0.3405	1.488	GCATTT
CUP9 (B)	8	0.3974	1.292	TGACACAT
DAL80 (B)	7	0.3849	1.567	CGATAAG
DAL81 (H)	19	0.6253	1.721	AAAAGCCGCGGGCGGGATT
DAL82 (B)	9	0.5643	1.043	AATGTGCGC
DAL82 (H)	7	0.3427	1.445	GATAAGA
DAL82 (M)	9	0.4414	0.984	AAAGATGCG
DIG1 (M)	12	0.3952	0.663	AAATATGAAACA
DOT6 (B)	5	0.5050	1.619	CGATG
ECM22 (B)	7	0.6429	1.331	CTCCGGA
ECM22 (M)	7	0.2973	1.740	GTATAAG
ECM22 (Z)	12	0.7252	1.033	CGGGTGGCCGGA
ECM23 (B)	6	0.3913	1.236	AGATCT
FHL1 (Z)	8	0.5438	1.326	GACGCAAA
FKH2 (B)	7	0.2679	1.922	GTAAACA
FKH2 (H)	11	0.2606	0.986	AAAAGTAAACA
FKH2 (Z)	8	0.2242	1.591	GTAAACAA

Continued on next page

TF (Study)	Length	GC content	Information	Canonical sequence
FZF1 (B)	6	0.3333	2.000	CTATCA
GAL4 (H)	17	0.5744	0.431	CGGAAAAATATACTCCG
GAL4 (M)	17	0.7258	0.967	CGGAGGACAGTGCCCCG
GAL4 (Z)	19	0.6601	0.692	TCGGG . . . . CCGA
GAT1 (H)	7	0.3158	1.552	AGATAAG
GAT1 (Z)	8	0.4218	1.498	CCTTATCG
GAT3 (B)	7	0.4056	1.046	AGATCTA
GAT3 (M)	8	0.2771	1.371	ATAACATG
GAT4 (Z)	8	0.3227	1.478	TAGATCTA
GCN4 (M)	8	0.4194	1.337	ATGACTCA
GCN4 (Z)	7	0.4203	1.609	TGAGTCA
GCR1 (H)	9	0.6585	1.622	GGCTTCC[A/T]C
GCR1 (M)	8	0.6821	1.474	GGGCTTCC
GCR2 (M)	7	0.5798	1.369	GCTTCCA
GIS1 (B)	7	0.6151	1.403	CCCCTAA
GLN3 (B)	5	0.2543	1.562	GATAA
GLN3 (H)	9	0.3223	1.129	GATAAGATA
GLN3 (M)	8	0.4293	0.980	AGATAAGA
GLN3 (Z)	13	0.3963	1.005	TTAGGCCTTATCA
GSM1 (Z)	9	0.5997	1.274	AAACTCCGG
GTS1 (M)	6	0.3558	1.527	TACCAA
GZF3 (H)	6	0.3416	1.720	GATAAG
GZF3 (Z)	8	0.3786	1.598	CTGATAAG
HAC1 (B)	7	0.5617	1.642	GACACGT
HAC1 (H)	11	0.6729	1.639	G[A/C]CAGCGTGTC
HAC1 (M)	10	0.5089	1.541	AGGACACGTA
HAL9 (B)	5	0.6721	1.713	CGGAA
HAP1 (H)	11	0.5372	1.086	GGAAATATCGG
HAP1 (M)	10	0.5625	0.807	CGATAACGCC
HAP2 (H)	5	0.4048	1.721	CCAAT
HAP2 (M)	8	0.4946	1.143	CCAATGAG
HAP4 (H)	9	0.4485	0.873	GACCAATCA
HAP4 (M)	8	0.4460	1.380	CCAATCAG
HCM1 (B)	8	0.2595	1.112	ATAAACAA

Continued on next page

TF (Study)	Length	GC content	Information	Canonical sequence
HMRA2 (B)	8	0.2991	1.341	CATGTAAT
HSF1 (B)	8	0.3984	1.272	ATGGAACA
HSF1 (H)	13	0.3715	0.704	TTCTATAACATTC
IME1 (M)	8	0.8579	1.672	CCGCCGAG
INO2 (H)	7	0.5757	1.325	CACATGC
INO2 (M)	9	0.4971	1.354	GCATGTGAA
INO4 (H)	8	0.4133	1.319	CATGTGAA
IXR1 (M)	15	0.6214	1.380	AAGCCGGAAGCGGGG
LEU3 (B)	10	0.7536	1.224	CCGTTACCGG
LEU3 (H)	10	0.7506	1.082	CCGGTACCGG
LEU3 (Z)	9	0.6505	1.165	ACCGGCGAA
LYS14 (B)	8	0.4757	1.447	CCGGAATT
LYS14 (Z)	8	0.3886	1.706	AAATTCCG
MAC1 (H)	7	0.4320	1.721	GAGCAAA
MAC1 (M)	9	0.4837	0.886	GAGCGAAAA
MATA1 (M)	7	0.3610	1.591	[A/G]CACAAT
MATALPHA2 (Z)	8	0.2635	1.566	AACAATAG
MBP1 (B)	6	0.7109	1.191	ACGCGT
MBP1 (H)	7	0.6417	1.327	GACGCGT
MBP1 (Z)	8	0.6266	1.494	ACGCGTCA
MCM1 (H)	12	0.4435	0.691	TTCCTAATTAGG
MCM1 (Z)	12	0.4647	0.732	TCCCAATTCGGA
MET28 (M)	6	0.6621	1.708	CTGTGG
MET31 (B)	9	0.6745	1.232	[A/G]GTGTGGCG
MET31 (H)	9	0.4471	1.721	AAACTGTGG
MET31 (M)	7	0.6041	1.419	GGTGTGG
MET32 (B)	7	0.6674	1.291	CGCCACA
MET32 (M)	9	0.5688	1.641	AACTGTGGC
MET32 (Z)	9	0.6486	1.513	AGTGTGGCG
MET4 (H)	13	0.4695	1.279	AAAAAATGTGGTG
MET4 (M)	8	0.5271	1.720	AACTGTGG
MGA1 (Z)	9	0.3672	1.194	ATAGAACAC
MIG1 (B)	6	0.8992	1.511	CCCCGC
MIG1 (M)	10	0.5494	1.229	AAAAG[C/T]GGGG

Continued on next page

TF (Study)	Length	GC content	Information	Canonical sequence
MIG1 (Z)	8	0.7577	1.489	ATGCGGGG
MIG2 (Z)	9	0.6774	1.433	CCCCGCAAT
MOT3 (H)	6	0.4738	1.343	[A/C/T]AGG[C/T]A
MSN2 (B)	5	0.8783	1.733	AGGGG
MSN2 (H)	9	0.7076	1.297	AAGGGGCGG
MSN2 (M)	7	0.7322	1.379	AAGGGGC
MSN4 (H)	6	0.7379	1.572	[A/C]AGGGG
NDD1 (H)	13	0.4831	0.827	TTTCCCAATTGGG
NDD1 (M)	10	0.4612	1.046	AAAGGGGAAA
NDT80 (Z)	9	0.3908	1.373	GCCACAAAA
NHP10 (B)	8	0.8500	1.490	GCCGGGGA
NHP6A (Z)	10	0.1542	0.892	CTATATATAA
NHP6B (Z)	12	0.1860	0.575	TATTTATATATA
NRG1 (H)	7	0.7019	1.490	GGACCCT
NRG1 (Z)	8	0.6600	1.570	CAGGGTCC
OAF1 (B)	8	0.5982	1.263	CGGAGATA
OAF1 (Z)	9	0.4490	1.121	TAAATCCGA
OPI1 (H)	7	0.5000	1.594	TCGAA[C/T]C
OPI1 (M)	6	0.6020	1.636	GAACCG
PBF2 (Z)	8	0.5923	1.426	CTCATCGC
PDR1 (B)	8	0.7917	1.251	TCCGCGGA
PDR1 (H)	8	0.5249	1.250	GCCGAATA
PDR1 (M)	9	0.6619	1.189	CCGCCGAAA
PDR3 (H)	8	0.7378	1.720	TCCGCGGA
PDR8 (B)	8	0.5721	1.536	[A/G]CGGAGAT
PHD1 (B)	7	0.5956	1.095	CCTGCAG
PHD1 (H)	8	0.7951	0.868	GCTGCAGG
PHD1 (M)	6	0.6442	1.271	AGGCAC
PHD1 (Z)	9	0.4736	1.118	CATGCATCA
PHO2 (B)	6	0.0141	1.226	ATAATA
PHO2 (H)	11	0.7657	1.320	CGTGCGGTGCG
PHO2 (M)	6	0.1725	1.328	ATTAAG
PHO2 (Z)	8	0.0733	0.973	ATTAATTA
PHO4 (B)	7	0.7383	1.048	GCGCGTG

Continued on next page

TF (Study)	Length	GC content	Information	Canonical sequence
PHO4 (H)	7	0.6518	1.321	CACGTGG
PHO4 (Z)	8	0.6863	1.364	GCACGTGC
PUT3 (B)	8	0.7943	1.312	CCCGGGAA
PUT3 (H)	16	0.6785	0.645	CGG . . . . CCG
PUT3 (M)	9	0.7589	1.721	CGGGAAGCC
PUT3 (Z)	8	0.7047	0.983	TCCCGGGA
RAP1 (B)	9	0.4864	1.310	ACCCATACA
RAP1 (M)	10	0.5890	1.175	ACCCATACAC
RAP1 (Z)	8	0.6003	1.296	GGGTGTAC
RDR1 (B)	8	0.6027	1.496	TGCGGAAC
RDR1 (Z)	8	0.5681	1.244	TGCGGATA
RDS1 (B)	7	0.9361	1.393	CGGCCG[A/C/G]
RDS1 (Z)	8	0.7921	1.620	TCGGCCGA
RDS2 (B)	7	0.7444	1.452	CTCGGGG
RDS2 (Z)	9	0.4640	1.225	AAAACCCGA
REB1 (B)	8	0.5250	1.668	GTTACCCG
REI1 (B)	7	0.7381	1.648	CCCCTGA
RFX1 (B)	8	0.4952	1.415	GGTTGC[C/T]A
RFX1 (H)	13	0.4651	1.195	TTGCCATGGCAAC
RGT1 (H)	7	0.5680	1.229	CGGA . . A
RGT1 (M)	8	0.3684	1.844	CGGAAAAA
RGT1 (Z)	9	0.4041	1.360	CGGAAAAAT
RIM101 (B)	7	0.6458	1.758	CGCCAAG
RIM101 (H)	7	0.5680	1.721	TGCCAAG
RLM1 (H)	10	0.2145	1.367	CTA[A/T][A/T][A/T][A/T]TAG
RLM1 (M)	9	0.2591	1.012	TATTTATAG
RLR1 (H)	11	0.2196	1.287	ATTTTCTTCTT
RLR1 (M)	13	0.3751	1.442	ACAGTACTTACAG
RME1 (M)	10	0.4143	0.960	TCCAAAGGAA
ROX1 (B)	5	0.2008	1.976	ACAAT
ROX1 (H)	9	0.3414	1.425	[C/T][C/G][C/T]ATTGTT
ROX1 (M)	9	0.6305	0.951	AAAAGCCCG
RPH1 (B)	8	0.5625	1.467	ACCCCTAA
RPH1 (H)	10	0.5952	1.721	CCCCTTAAGG

Continued on next page

TF (Study)	Length	GC content	Information	Canonical sequence
RPH1 (Z)	8	0.4977	1.333	ATTAGGGG
RPN4 (B)	7	0.8078	1.633	GGTGGCG
RPN4 (H)	8	0.6124	1.599	TTGCCACC
RPN4 (M)	9	0.5944	1.543	GGTGGCAAA
RPN4 (Z)	8	0.8655	1.689	CGCCACCC
RSC3 (B)	5	0.9364	1.531	CGCGC
RSC3 (Z)	12	0.7270	0.971	CGCGCGCTCTGT
RSC30 (B)	8	0.9423	1.260	CGCGCGCG
RSC30 (Z)	10	0.9026	1.115	CGCGCGCGCG
RTG3 (H)	6	0.6584	1.720	GGTCAC
RTG3 (M)	8	0.5221	1.291	ATGACGCA
RTG3 (Z)	8	0.6791	1.391	GCACGTGC
SFL1 (M)	8	0.5000	1.708	GAAGCTTC
SFL1 (Z)	9	0.3694	0.976	ATAGAAGAA
SFP1 (M)	9	0.5466	1.264	ATGTACGGG
SFP1 (Z)	9	0.0822	1.172	AAAAATTTT
SIG1 (B)	5	0.0103	1.827	ATATA
SIG1 (H)	12	0.3021	1.247	AGGAA[A/T]CAAAAA
SIP4 (B)	7	0.7016	1.241	CTCCGGA
SIP4 (H)	11	0.5428	1.119	CGGATTAATGG
SIP4 (Z)	8	0.5761	1.214	TTCCGGAA
SKN7 (B)	5	0.9012	1.557	GGCCA
SKN7 (H)	9	0.8154	0.712	GCCTGGGCC
SKN7 (M)	8	0.8377	0.955	GCCGGGCC
SKN7 (Z)	16	0.4965	0.461	AGTTATGGCCATATCT
SKO1 (H)	6	0.5000	1.721	ACGTCA
SKO1 (M)	7	0.4761	1.353	TACGTCA
SMP1 (H)	14	0.2281	1.468	ACTACTA[A/T][A/T][A/T][A/T]TAG
SMP1 (Z)	10	0.2199	0.715	CTATAATTAA
SNF1 (M)	15	0.6573	1.808	GGGGATCGAACCCGG
SNT2 (H)	9	0.6591	1.203	GGCGCTATC
SOK2 (B)	9	0.5326	0.881	ACCTGCAGG
SOK2 (H)	8	0.5737	0.817	TGCAGGAA
SOK2 (M)	8	0.5875	1.096	GCAGGCAA

Continued on next page

TF (Study)	Length	GC content	Information	Canonical sequence
SPT15 (Z)	10	0.1534	1.260	GATATATATA
SPT2 (H)	10	0.3212	1.128	CCTGTATTTA
SPT2 (M)	10	0.2531	1.086	AATTAAAGAA
SPT23 (H)	8	0.1951	1.212	AAAATCAA
SRD1 (Z)	8	0.4117	1.696	AGATCTAC
STB1 (H)	9	0.5186	0.868	AAACGCGAA
STB1 (M)	8	0.5211	1.463	CGCGAAAA
STB2 (M)	8	0.6058	1.364	CGGGTAAG
STB3 (Z)	9	0.1468	1.491	AATTTTTCA
STB4 (H)	9	0.5947	1.192	TCGG[A/T][A/T]CGA
STB5 (B)	8	0.5856	1.072	CGGTGTTA
STB5 (H)	9	0.4581	1.149	CGGTGTTAT
STB5 (M)	10	0.5053	0.947	CGGTGTTATA
STE12 (B)	7	0.3095	1.867	TGAAACA
STP1 (H)	12	0.8173	1.216	CGGC . . .[A/G]CGGC
STP1 (M)	8	0.8396	1.279	TGCGGCGG
STP2 (Z)	8	0.7897	1.246	GCGCCGCA
STP3 (B)	9	0.5964	1.220	GCTAGCGCA
STP4 (M)	14	0.8355	1.457	CGCCGTGGGACGCG
STP4 (Z)	10	0.6270	1.096	GTGCGGCTGA
SUM1 (B)	8	0.0700	1.127	AAATTTTT
SUM1 (H)	9	0.4243	0.818	TGTCAGAAA
SUM1 (Z)	8	0.0154	1.079	AATTAATT
SUT1 (H)	10	0.8387	0.694	GCGGGGCCGG
SUT1 (M)	7	0.8536	1.021	CGCGGGG
SUT2 (Z)	8	0.5272	1.644	AACTCCGA
SWI4 (B)	8	0.5337	1.492	ACGCGAAA
SWI5 (B)	8	0.6202	1.423	TGCTGGTT
SWI5 (H)	6	0.6586	1.425	[G/T]GCTG[A/G]
SWI5 (M)	6	0.7152	1.050	TGCTGG
SWI6 (M)	6	0.7675	1.425	GACGCG
TBF1 (B)	7	0.5349	1.246	AACCCTA
TBF1 (Z)	8	0.4219	1.651	AACCCTAA
TBS1 (Z)	8	0.7561	1.706	CGGATCCG

Continued on next page

TF (Study)	Length	GC content	Information	Canonical sequence
TEA1 (B)	8	0.7062	1.166	GCGG[A/G]CAT
TEA1 (Z)	15	0.3749	1.239	CGGAAATTAAATACC
TEC1 (H)	7	0.3790	1.267	AAGAATG
TEC1 (M)	7	0.4341	1.400	ACATTCT
TEC1 (Z)	8	0.3856	1.451	ACATTCTT
THI2 (H)	12	0.3359	0.898	GAAACTCTAAGA
TOS8 (B)	8	0.4111	1.288	CTGTCAAA
TYE7 (B)	7	0.5857	1.700	CACGTGA
TYE7 (Z)	8	0.5265	1.361	TCACGTGA
UGA3 (B)	8	0.8281	1.371	CGGCGGGA
UGA3 (H)	10	0.7856	1.032	CCG . . . . CGG
UME1 (H)	11	0.2669	1.240	AAGGAAAAGTA
UME6 (B)	8	0.7422	1.159	CGCGGC[A/T]A
UME6 (M)	9	0.7749	1.397	AGCCGCCGA
UME6 (Z)	8	0.7109	1.439	GGCGGCTA
UPC2 (B)	6	0.4080	1.420	ATACGA
USV1 (Z)	8	0.6231	1.703	CCCCTGAA
XBP1 (B)	6	0.5248	1.482	CTCGAG
XBP1 (H)	7	0.5680	1.721	CTTCGAG
XBP1 (M)	8	0.6270	1.346	CTCGAGGA
XBP1 (Z)	8	0.5310	1.224	TCTCGAGA
YAP1 (H)	8	0.3250	1.435	TTAGTCAG
YAP1 (M)	9	0.4232	1.152	GCTGACTAA
YAP3 (B)	8	0.3317	1.552	ATTACGTA
YAP5 (M)	6	0.3216	1.344	AAGCAT
YAP6 (M)	8	0.2860	1.160	TTACATAA
YAP6 (Z)	10	0.3454	0.890	GATTACGTAA
YAP7 (H)	8	0.2395	1.099	ATTAGTAA
YBL054W (B)	8	0.6875	1.367	CGATGCCC
YBR239C (B)	8	0.5662	1.427	ACCGGAAC
YDR026C (H)	8	0.6427	1.843	TTACCCGG
YDR026C (M)	6	0.8331	1.992	ACCCGG
YDR520C (M)	9	0.8800	1.561	CTCCGGCGG
YDR520C (Z)	8	0.4968	1.758	CGGAGATA

Continued on next page

TF (Study)	Length	GC content	Information	Canonical sequence
YER051W (M)	6	0.3004	1.787	TTCGAA
YER130C (Z)	8	0.5021	1.646	AATAGGGG
YER184C (B)	8	0.6635	1.157	CTCCGGAA
YGR067C (Z)	8	0.7498	1.507	GCGGGGTA
YHP1 (H)	6	0.1828	1.720	TAATTG
YJL103C (B)	8	0.7300	0.958	CTCCGGAG
YKL222C (B)	8	0.5208	1.284	ACGGAAAT
YKL222C (Z)	8	0.4764	1.292	CGGAAATA
YLL054C (B)	7	0.8061	1.220	CGGCCGA
YLL054C (Z)	18	0.6200	0.554	TCCGT . . . . CGGA
YLR278C (B)	8	0.5915	1.322	CCGGAGTT
YML081W (B)	7	0.7921	1.543	CCCCGCA
YML081W (M)	9	0.4941	1.648	CCAGTCTGA
YML081W (Z)	8	0.7449	1.516	ACCCCGCA
YNR063W (Z)	8	0.5062	1.494	ATCTCCGA
YOX1 (H)	10	0.2621	1.459	A[C/G]AATA . TGA
YOX1 (M)	19	0.4060	1.441	ATTACGTTTCCTAAAAGGG
YOX1 (Z)	8	0.1038	1.378	TTAATTAA
YPR013C (B)	8	0.4492	1.332	TGTAGATC
YPR015C (Z)	8	0.3621	1.708	CGTAAATC
YPR022C (B)	6	0.7760	1.789	CCCCAC
YPR196W (Z)	9	0.4423	0.949	ATTTCTCCG
YRR1 (B)	9	0.4627	1.070	TTATCTCCG
YRR1 (M)	11	0.4248	1.379	TTTTGTTACCC
YRR1 (Z)	9	0.3695	1.515	CGGAAATAA
ZAP1 (H)	14	0.4331	1.625	ACCCT[A/C]AAGGTT[A/G]T
ZAP1 (M)	11	0.4755	1.683	ACCTTAAAGGT
ZMS1 (B)	9	0.6889	1.156	TTCCCCGCA
