# Supplementary material for: Redundancy and the Evolution of Cis-Regulatory Element Multiplicity
Source: PLoS Comput Biol. 2010 Jul 8;6(7):e1000848. doi: 10.1371/journal.pcbi.1000848 (PMC2900288; doi:10.1371/journal.pcbi.1000848)
Supplement: Table S2 — Binding site PWM models not considered in our study. These PWMs were excluded because they were almost identical to the PWMs listed in the ‘Equivalent’ column, shown in Table S1. Letters in parentheses after TF names indicate the study from which we took the PWM data: B, Badis et al. (2008); H, Harbison et al. (2004); M, MacIsaac et al. (2006); Zhu et al. (2009). (0.04 MB PDF) [file pcbi.1000848.s007.pdf]

Table S2. Binding site PWM models not considered in our study. These PWMs were excluded because they were almost identical to the PWMs listed in the 'Equivalent' column, shown in Table S1. Letters in parentheses after TF names indicate the study from which we took the PWM data: B, Badis *et al.* (2008); H, Harbison *et al.* (2004); M, MacIsaac *et al.* (2006); Zhu *et al.* (2009).

TF (Study)	Equivalent
ACE2 (H)	SWI5 (B)
ADR1 (B)	YPR022C (B)
ADR1 (M)	ADR1 (H)
CBF1 (H)	TYE7 (B)
CIN5 (B)	YAP6 (Z)
CUP9 (Z)	CUP9 (B)
DAL80 (H)	GLN3 (B)
DAL80 (M)	GLN3 (B)
DAL81 (M)	DAL81 (H)
DIG1 (H)	STE12 (B)
EDS1 (B)	RGT1 (Z)
FHL1 (B)	FHL1 (Z)
FHL1 (H)	RAP1 (B)
FHL1 (M)	RAP1 (B)
FKH1 (H)	FKH2 (Z)
FKH1 (M)	FKH2 (Z)
FKH1 (Z)	FKH2 (Z)
FKH2 (M)	FKH2 (H)
GAL80 (H)	GAL4 (H)
GAL80 (M)	GAL4 (H)
GAT1 (B)	GZF3 (Z)
GAT1 (M)	GAT1 (H)
GAT3 (Z)	SRD1 (Z)
GAT4 (B)	ECM23 (B)
GCN4 (H)	GCN4 (Z)
GZF3 (B)	DAL80 (B)
GZF3 (M)	GZF3 (H)
HAL9 (Z)	YLL054C (Z)
HAP1 (B)	YKL222C (Z)

Continued on next page

TF (Study)	Equivalent
HAP3 (H)	HAP2 (H)
HAP3 (M)	HAP2 (H)
HAP5 (H)	HAP2 (H)
HAP5 (M)	HAP2 (H)
HSF1 (M)	HSF1 (H)
INO4 (M)	INO4 (H)
LEU3 (M)	LEU3 (H)
MBP1 (M)	MBP1 (H)
MCM1 (M)	NDD1 (H)
MET32 (H)	MET31 (H)
MIG2 (B)	MIG1 (B)
MIG3 (B)	MIG1 (B)
MIG3 (Z)	YML081W (Z)
MOT3 (M)	MOT3 (H)
MSN4 (B)	MSN2 (B)
MSN4 (M)	MSN2 (M)
NRG1 (M)	NRG1 (H)
PBF1 (Z)	PBF2 (Z)
PDR1 (Z)	SIP4 (Z)
PDR3 (M)	PDR3 (H)
PHO4 (M)	PHO4 (H)
RAP1 (H)	RAP1 (B)
RCS1 (H)	AFT1 (Z)
RCS1 (M)	AFT2 (M)
RDS1 (H)	RDS1 (Z)
RDS1 (M)	RDS1 (B)
REB1 (H)	STB2 (M)
REB1 (M)	YDR026C (H)
RFX1 (M)	RFX1 (H)
RGM1 (B)	MSN2 (B)
RGT1 (B)	RGT1 (Z)
RIM101 (M)	RIM101 (H)
RPH1 (M)	RPH1 (H)
SFP1 (H)	RAP1 (B)

Continued on next page

TF (Study)	Equivalent
SIP4 (M)	SIP4 (H)
SMP1 (M)	SMP1 (H)
SNT2 (M)	SNT2 (H)
SPT23 (M)	SPT23 (H)
SRD1 (B)	GAT3 (B)
STB4 (B)	CEP3 (B)
STB4 (M)	STB4 (H)
STE12 (H)	STE12 (B)
STE12 (M)	STE12 (B)
STP4 (B)	STP3 (B)
SUM1 (M)	SUM1 (H)
SWI4 (H)	SWI4 (B)
SWI4 (M)	SWI4 (B)
SWI6 (H)	SWI4 (B)
TBS1 (B)	TBS1 (Z)
TEC1 (B)	TEC1 (Z)
THI2 (M)	THI2 (H)
TYE7 (H)	TYE7 (Z)
TYE7 (M)	CBF1 (M)
UGA3 (M)	UGA3 (H)
UME6 (H)	UME6 (Z)
YAP1 (Z)	CIN5 (H)
YAP3 (H)	ARR1 (H)
YAP3 (M)	ARR1 (H)
YAP5 (H)	ARR1 (H)
YAP6 (H)	ARR1 (H)
YAP7 (M)	YAP7 (H)
YBR239C (Z)	SIP4 (Z)
YDR520C (B)	YKL222C (Z)
YER130C (B)	YER130C (Z)
YGR067C (B)	MIG1 (B)
YHP1 (M)	YHP1 (H)
YNR063W (B)	YNR063W (Z)
YOX1 (B)	YOX1 (Z)

Continued on next page

TF (Study)	Equivalent
YPL230W (B)	MSN2 (B)
YPR013C (Z)	YPR015C (Z)
YPR196W (B)	YPR196W (Z)
YRM1 (B)	YKL222C (B)
YRM1 (Z)	YKL222C (Z)
