# Supplementary material for: Redundancy and the Evolution of Cis-Regulatory Element Multiplicity
Source: PLoS Comput Biol. 2010 Jul 8;6(7):e1000848. doi: 10.1371/journal.pcbi.1000848 (PMC2900288; doi:10.1371/journal.pcbi.1000848)
Supplement: Table S3 — Data used to construct Figure 6. The data are sorted by decreasing M Obs/M Exp. The second and third columns show the Total number of binding sites revealed in a scan across the number of promoter regions in the ‘Prom’ column (the numbers vary because the minimum promoter length considered in each scan is twice the length of the PWM). Letters in parentheses after TF names indicate the study from which we took the PWM data: B, Badis et al. (2008); H, Harbison et al. (2004); M, MacIsaac et al. (2006); Zhu et al. (2009). (0.05 MB PDF) [file pcbi.1000848.s008.pdf]

Table S3. Data used to construct Figure 6. The data are sorted by decreasing $M_{\mathrm{Obs}}/M_{\mathrm{Exp}}$. The second and third columns show the Total number of binding sites revealed in a scan across the number of promoter regions in the 'Prom' column (the numbers vary because the minimum promoter length considered in each scan is twice the length of the PWM). Letters in parentheses after TF names indicate the study from which we took the PWM data: B, Badis *et al.* (2008); H, Harbison *et al.* (2004); M, MacIsaac *et al.* (2006); Zhu *et al.* (2009).

TF (Study)	Total	Prom	M_{Obs}	M_{Exp}	$M_{\mathrm{Obs}}/M_{\mathrm{Exp}}$
ZAP1 (H)	2	3007	1.0000	0.0003	3007.427
RSC30 (Z)	3	3020	0.5000	0.0005	1006.881
ZAP1 (M)	3	3020	0.5000	0.0005	1006.881
SIP4 (H)	5	3020	0.2500	0.0008	302.107
INO2 (M)	9	3021	0.2857	0.0015	191.932
ASH1 (M)	8	3020	0.1429	0.0013	107.918
YAP6 (Z)	17	3020	0.2143	0.0028	76.226
MCM1 (H)	13	3014	0.0909	0.0022	42.193
RSC30 (B)	68	3023	0.3830	0.0112	34.216
PDR1 (M)	16	3021	0.0667	0.0026	25.204
LEU3 (B)	16	3020	0.0667	0.0026	25.195
CBF1 (M)	43	3023	0.1667	0.0071	23.506
XBP1 (M)	48	3023	0.1707	0.0079	21.579
MBP1 (Z)	31	3023	0.1071	0.0051	20.942
NDD1 (H)	18	3010	0.0588	0.0030	19.698
PUT3 (B)	20	3023	0.0526	0.0033	15.933
SMP1 (Z)	37	3020	0.0882	0.0061	14.442
INO4 (H)	30	3023	0.0714	0.0050	14.426
GAT3 (M)	44	3023	0.1000	0.0073	13.784
MET4 (M)	42	3023	0.0769	0.0069	11.106
YLL054C (B)	24	3025	0.0435	0.0040	10.979
SKN7 (H)	66	3021	0.1071	0.0109	9.855
RDS2 (B)	47	3025	0.0682	0.0077	8.806
UME6 (M)	28	3021	0.0385	0.0046	8.316
RPN4 (B)	28	3025	0.0370	0.0046	8.019
PHO4 (B)	148	3025	0.1901	0.0242	7.853
UGA3 (H)	72	3020	0.0909	0.0119	7.665

Continued on next page

TF (Study)	Total	Prom	M_{Obs}	M_{Exp}	$M_{\text{Obs}}/M_{\text{Exp}}$
SUT1 (M)	41	3025	0.0513	0.0068	7.589
RPH1 (Z)	41	3023	0.0513	0.0068	7.584
UME6 (B)	42	3023	0.0500	0.0069	7.219
SKN7 (M)	42	3023	0.0500	0.0069	7.219
YBL054W (B)	52	3023	0.0612	0.0086	7.145
INO2 (H)	68	3025	0.0794	0.0112	7.095
STP1 (M)	88	3023	0.1013	0.0145	7.001
HSF1 (H)	43	3010	0.0488	0.0071	6.850
RDS1 (B)	44	3025	0.0476	0.0073	6.568
GAT1 (Z)	55	3023	0.0588	0.0091	6.492
SWI6 (M)	198	3027	0.2013	0.0322	6.241
LYS14 (B)	73	3023	0.0735	0.0120	6.122
YBR239C (B)	32	3023	0.0323	0.0053	6.109
STB4 (H)	32	3021	0.0323	0.0053	6.105
RGT1 (Z)	82	3021	0.0822	0.0135	6.092
SFP1 (Z)	317	3021	0.2924	0.0513	5.701
IME1 (M)	34	3023	0.0312	0.0056	5.570
MET31 (B)	48	3021	0.0435	0.0079	5.492
CHA4 (B)	34	3023	0.0303	0.0056	5.402
TYE7 (B)	94	3025	0.0805	0.0154	5.213
YPR022C (B)	168	3027	0.1370	0.0274	4.996
CBF1 (B)	72	3023	0.0588	0.0118	4.965
FKH2 (H)	36	3020	0.0286	0.0059	4.806
PUT3 (H)	63	3001	0.0500	0.0104	4.785
STB1 (M)	83	3023	0.0641	0.0136	4.697
CEP3 (Z)	74	3023	0.0571	0.0122	4.693
ACE2 (M)	52	3023	0.0400	0.0086	4.668
YPR196W (Z)	75	3021	0.0571	0.0123	4.628
YER184C (B)	37	3023	0.0278	0.0061	4.551
SFP1 (M)	65	3021	0.0484	0.0107	4.519
MET32 (B)	128	3025	0.0940	0.0210	4.484
PHO2 (Z)	258	3023	0.1831	0.0419	4.371
MBP1 (H)	127	3025	0.0870	0.0208	4.180
HAP4 (M)	55	3023	0.0377	0.0091	4.164

Continued on next page

TF (Study)	Total	Prom	M_{Obs}	M_{Exp}	$M_{\text{Obs}}/M_{\text{Exp}}$
UME6 (Z)	79	3023	0.0533	0.0130	4.105
HAC1 (B)	39	3025	0.0263	0.0064	4.094
NDD1 (M)	70	3020	0.0448	0.0115	3.883
FKH2 (Z)	101	3023	0.0632	0.0166	3.808
SWI4 (B)	71	3023	0.0441	0.0117	3.776
AFT2 (M)	93	3025	0.0575	0.0153	3.764
RPH1 (B)	58	3023	0.0357	0.0096	3.738
DAL82 (B)	58	3021	0.0357	0.0096	3.736
DAL80 (B)	72	3025	0.0441	0.0118	3.726
LYS14 (Z)	41	3023	0.0250	0.0068	3.697
STP2 (Z)	41	3023	0.0250	0.0068	3.697
SPT15 (Z)	162	3020	0.0946	0.0265	3.568
TEA1 (B)	42	3023	0.0244	0.0069	3.522
PDR1 (H)	60	3023	0.0345	0.0099	3.490
SWI5 (B)	74	3023	0.0423	0.0122	3.470
YAP3 (B)	146	3023	0.0821	0.0239	3.435
BAS1 (M)	153	3025	0.0851	0.0250	3.402
MAC1 (M)	61	3021	0.0339	0.0101	3.372
STB5 (B)	125	3023	0.0684	0.0205	3.337
MIG1 (B)	319	3027	0.1698	0.0515	3.297
GLN3 (H)	63	3021	0.0333	0.0104	3.211
YOX1 (H)	44	3020	0.0233	0.0073	3.202
ASG1 (B)	393	3027	0.2013	0.0631	3.189
NHP6B (Z)	716	3014	0.3571	0.1128	3.167
BAS1 (H)	234	3027	0.1148	0.0380	3.021
RGT1 (M)	166	3023	0.0811	0.0271	2.988
GAT3 (B)	93	3025	0.0455	0.0153	2.977
STB1 (H)	65	3021	0.0317	0.0107	2.965
AFT2 (B)	46	3023	0.0222	0.0076	2.930
FHL1 (Z)	47	3023	0.0217	0.0077	2.806
RDR1 (B)	67	3023	0.0308	0.0110	2.790
TBF1 (B)	118	3025	0.0536	0.0193	2.770
MET31 (M)	118	3025	0.0536	0.0193	2.770
STE12 (B)	218	3025	0.0969	0.0355	2.733

Continued on next page

TF (Study)	Total	Prom	M_{Obs}	M_{Exp}	$M_{\text{Obs}}/M_{\text{Exp}}$
OAF1 (Z)	48	3021	0.0213	0.0079	2.687
RTG3 (H)	130	3027	0.0569	0.0213	2.675
CRZ1 (M)	110	3025	0.0481	0.0180	2.665
YML081W (B)	361	3025	0.1546	0.0581	2.659
CRZ1 (B)	69	3021	0.0299	0.0114	2.627
CIN5 (M)	100	3021	0.0421	0.0164	2.562
PHD1 (H)	86	3023	0.0361	0.0141	2.557
PHO4 (H)	70	3025	0.0294	0.0115	2.555
MET28 (M)	199	3027	0.0824	0.0324	2.543
CUP9 (B)	87	3023	0.0357	0.0143	2.497
DAL82 (H)	172	3025	0.0688	0.0281	2.448
MSN2 (M)	181	3025	0.0719	0.0295	2.433
YKL222C (Z)	115	3023	0.0455	0.0189	2.409
HSF1 (B)	89	3023	0.0349	0.0146	2.385
ARG81 (M)	129	3023	0.0488	0.0211	2.307
PHD1 (M)	382	3027	0.1402	0.0614	2.284
AFT2 (H)	348	3027	0.1275	0.0561	2.273
CEP3 (B)	170	3025	0.0625	0.0278	2.251
YAP7 (H)	212	3023	0.0761	0.0345	2.205
XBP1 (Z)	53	3023	0.0192	0.0087	2.202
GAT1 (H)	183	3025	0.0643	0.0299	2.155
RDR1 (Z)	77	3023	0.0267	0.0127	2.105
RLM1 (M)	124	3021	0.0424	0.0203	2.083
GCN4 (Z)	79	3025	0.0260	0.0130	2.000
RSC3 (B)	583	3033	0.1824	0.0921	1.979
RTG3 (M)	98	3023	0.0316	0.0161	1.962
STB3 (Z)	330	3021	0.1044	0.0533	1.957
ARR1 (H)	276	3025	0.0866	0.0447	1.936
DIG1 (M)	57	3014	0.0179	0.0094	1.896
MBP1 (B)	566	3027	0.1696	0.0897	1.889
TEC1 (Z)	146	3023	0.0429	0.0239	1.793
SUM1 (H)	190	3021	0.0556	0.0310	1.791
SPT2 (M)	103	3020	0.0303	0.0169	1.790
GCR2 (M)	260	3025	0.0750	0.0422	1.778

Continued on next page

TF (Study)	Total	Prom	M_{Obs}	M_{Exp}	$M_{\text{Obs}}/M_{\text{Exp}}$
RME1 (M)	104	3020	0.0300	0.0171	1.755
SKO1 (H)	290	3027	0.0821	0.0469	1.750
SWI5 (M)	345	3027	0.0971	0.0556	1.746
ACE2 (B)	161	3025	0.0455	0.0263	1.728
NRG1 (H)	60	3025	0.0169	0.0099	1.716
GZF3 (H)	479	3027	0.1280	0.0764	1.675
ROX1 (H)	164	3021	0.0449	0.0268	1.673
SOK2 (H)	123	3023	0.0336	0.0202	1.667
HCM1 (B)	299	3023	0.0797	0.0484	1.647
ECM23 (B)	221	3027	0.0580	0.0359	1.613
TEC1 (M)	559	3025	0.1426	0.0887	1.606
PHD1 (B)	632	3025	0.1581	0.0998	1.584
HMRA2 (B)	180	3023	0.0465	0.0294	1.582
DAL82 (M)	142	3021	0.0365	0.0233	1.569
CAT8 (B)	535	3033	0.1327	0.0849	1.564
SKN7 (B)	863	3033	0.2081	0.1336	1.557
HAP2 (M)	63	3023	0.0161	0.0104	1.555
SKO1 (M)	90	3025	0.0227	0.0148	1.538
ECM22 (M)	144	3025	0.0360	0.0236	1.527
SUM1 (Z)	428	3023	0.1047	0.0686	1.525
MSN2 (B)	828	3033	0.1958	0.1285	1.523
SIP4 (B)	64	3025	0.0159	0.0105	1.507
FZF1 (B)	482	3027	0.1093	0.0769	1.421
TOS8 (B)	150	3023	0.0347	0.0245	1.415
MSN4 (H)	459	3027	0.1029	0.0734	1.403
XBP1 (B)	492	3027	0.1098	0.0784	1.400
ARG80 (M)	621	3027	0.1336	0.0981	1.362
CST6 (M)	824	3027	0.1742	0.1282	1.359
YDR026C (M)	230	3027	0.0507	0.0374	1.356
SUM1 (B)	1452	3023	0.2923	0.2158	1.354
FKH2 (B)	232	3025	0.0507	0.0377	1.344
YAP1 (H)	68	3023	0.0149	0.0112	1.333
HAL9 (B)	1367	3033	0.2705	0.2038	1.327
ASH1 (H)	652	3027	0.1360	0.1027	1.323

Continued on next page

TF (Study)	Total	Prom	M_{Obs}	M_{Exp}	$M_{\text{Obs}}/M_{\text{Exp}}$
YHP1 (H)	849	3027	0.1731	0.1318	1.313
AZF1 (B)	1760	3025	0.3325	0.2553	1.302
RGT1 (H)	1146	3025	0.2230	0.1742	1.280
GTS1 (M)	557	3027	0.1120	0.0884	1.267
YPR013C (B)	143	3023	0.0288	0.0234	1.229
YLR278C (B)	71	3023	0.0143	0.0117	1.223
ADR1 (H)	2578	3033	0.4282	0.3504	1.222
SPT23 (H)	937	3023	0.1765	0.1447	1.219
YAP5 (M)	1719	3027	0.3016	0.2500	1.206
GLN3 (M)	103	3023	0.0200	0.0169	1.183
UPC2 (B)	1167	3027	0.2084	0.1770	1.178
NHP6A (Z)	146	3020	0.0282	0.0239	1.178
HAP2 (H)	1496	3033	0.2591	0.2209	1.173
TEC1 (H)	348	3025	0.0646	0.0561	1.152
OPI1 (M)	450	3027	0.0825	0.0720	1.147
MATALPHA2 (Z)	128	3023	0.0240	0.0210	1.144
YER051W (M)	313	3027	0.0578	0.0506	1.144
PHO2 (M)	2409	3027	0.3781	0.3324	1.137
SWI5 (H)	899	3027	0.1569	0.1391	1.128
ECM22 (B)	74	3025	0.0137	0.0122	1.126
MOT3 (H)	2265	3027	0.3541	0.3160	1.121
UME1 (H)	106	3020	0.0192	0.0174	1.104
PHO2 (B)	4641	3027	0.5936	0.5406	1.098
GLN3 (B)	2949	3033	0.4253	0.3897	1.091
MATA1 (M)	278	3025	0.0491	0.0450	1.089
DOT6 (B)	4807	3033	0.5940	0.5520	1.076
ROX1 (B)	3262	3033	0.4491	0.4209	1.067
YAP6 (M)	467	3023	0.0789	0.0747	1.056
SIG1 (B)	7380	3033	0.7280	0.7045	1.033
CIN5 (H)	177	3023	0.0292	0.0289	1.011
MAC1 (H)	158	3025	0.0260	0.0258	1.006
RIM101 (H)	112	3025	0.0183	0.0184	0.999
RFX1 (B)	180	3023	0.0286	0.0294	0.972
RIM101 (B)	164	3025	0.0252	0.0268	0.939

Continued on next page

TF (Study)	Total	Prom	M_{Obs}	M_{Exp}	$M_{\text{Obs}}/M_{\text{Exp}}$
ABF2 (B)	164	3027	0.0250	0.0268	0.934
ARR1 (M)	637	3023	0.0936	0.1006	0.930
GIS1 (B)	185	3025	0.0279	0.0302	0.926
CHA4 (M)	116	3023	0.0175	0.0190	0.922
SFL1 (Z)	83	3021	0.0122	0.0137	0.893
XBP1 (H)	93	3025	0.0109	0.0153	0.712
SOK2 (M)	96	3023	0.0105	0.0158	0.667
YDR026C (H)	101	3023	0.0100	0.0166	0.603
REB1 (B)	233	3023	0.0175	0.0379	0.461
STB2 (M)	268	3023	0.0152	0.0435	0.348
NRG1 (Z)	38	3023	0.0000	0.0063	0.000
RPN4 (Z)	6	3023	0.0000	0.0010	0.000
SIP4 (Z)	14	3023	0.0000	0.0023	0.000
YOX1 (Z)	55	3023	0.0000	0.0091	0.000
YPR015C (Z)	35	3023	0.0000	0.0058	0.000
LEU3 (Z)	2	3021	0.0000	0.0003	0.000
PBF2 (Z)	88	3023	0.0000	0.0145	0.000
YLL054C (Z)	2	2996	0.0000	0.0003	0.000
YER130C (Z)	18	3023	0.0000	0.0030	0.000
RSC3 (Z)	5	3014	0.0000	0.0008	0.000
YML081W (Z)	13	3023	0.0000	0.0021	0.000
MIG1 (Z)	9	3023	0.0000	0.0015	0.000
YNR063W (Z)	14	3023	0.0000	0.0023	0.000
YGR067C (Z)	16	3023	0.0000	0.0026	0.000
RDS1 (Z)	7	3023	0.0000	0.0012	0.000
SUT2 (Z)	21	3023	0.0000	0.0035	0.000
TBF1 (Z)	26	3023	0.0000	0.0043	0.000
CHA4 (Z)	24	3021	0.0000	0.0040	0.000
GSM1 (Z)	18	3021	0.0000	0.0030	0.000
RDS2 (Z)	26	3021	0.0000	0.0043	0.000
SRD1 (Z)	32	3023	0.0000	0.0053	0.000
ECM22 (Z)	2	3014	0.0000	0.0003	0.000
TBS1 (Z)	6	3023	0.0000	0.0010	0.000
CBF1 (Z)	7	3020	0.0000	0.0012	0.000

Continued on next page

TF (Study)	Total	Prom	M_{Obs}	M_{Exp}	$M_{\text{Obs}}/M_{\text{Exp}}$
YRR1 (Z)	15	3021	0.0000	0.0025	0.000
RAP1 (Z)	11	3023	0.0000	0.0018	0.000
GAL4 (Z)	7	2995	0.0000	0.0012	0.000
YDR520C (Z)	24	3023	0.0000	0.0040	0.000
TYE7 (Z)	28	3023	0.0000	0.0046	0.000
ARO80 (Z)	39	3023	0.0000	0.0064	0.000
PHO4 (Z)	15	3023	0.0000	0.0025	0.000
PUT3 (Z)	2	3023	0.0000	0.0003	0.000
GAT4 (Z)	12	3023	0.0000	0.0020	0.000
MGA1 (Z)	58	3021	0.0000	0.0096	0.000
USV1 (Z)	21	3023	0.0000	0.0035	0.000
AFT1 (Z)	8	3021	0.0000	0.0013	0.000
MET32 (Z)	19	3021	0.0000	0.0031	0.000
NDT80 (Z)	44	3021	0.0000	0.0073	0.000
GZF3 (Z)	26	3023	0.0000	0.0043	0.000
MIG2 (Z)	11	3021	0.0000	0.0018	0.000
PHD1 (Z)	22	3021	0.0000	0.0036	0.000
STP4 (Z)	5	3020	0.0000	0.0008	0.000
BAS1 (Z)	5	3021	0.0000	0.0008	0.000
RTG3 (Z)	7	3023	0.0000	0.0012	0.000
ABF1 (B)	55	3007	0.0000	0.0091	0.000
CST6 (B)	87	3023	0.0000	0.0143	0.000
NHP10 (B)	9	3023	0.0000	0.0015	0.000
OAF1 (B)	31	3023	0.0000	0.0051	0.000
PDR1 (B)	25	3023	0.0000	0.0041	0.000
PDR8 (B)	55	3023	0.0000	0.0091	0.000
REI1 (B)	45	3025	0.0000	0.0074	0.000
SOK2 (B)	29	3021	0.0000	0.0048	0.000
STP3 (B)	22	3021	0.0000	0.0036	0.000
UGA3 (B)	29	3023	0.0000	0.0048	0.000
YJL103C (B)	26	3023	0.0000	0.0043	0.000
YKL222C (B)	60	3023	0.0000	0.0099	0.000
YRR1 (B)	40	3021	0.0000	0.0066	0.000
ZMS1 (B)	26	3021	0.0000	0.0043	0.000

Continued on next page

TF (Study)	Total	Prom	M_{Obs}	M_{Exp}	$M_{\text{Obs}}/M_{\text{Exp}}$
RAP1 (B)	32	3021	0.0000	0.0053	0.000
ABF1 (H)	50	3014	0.0000	0.0083	0.000
AZF1 (H)	29	3005	0.0000	0.0048	0.000
CAD1 (H)	23	3021	0.0000	0.0038	0.000
GAL4 (H)	2	2999	0.0000	0.0003	0.000
GCR1 (H)	8	3021	0.0000	0.0013	0.000
HAP1 (H)	4	3020	0.0000	0.0007	0.000
HAP4 (H)	38	3021	0.0000	0.0063	0.000
LEU3 (H)	5	3020	0.0000	0.0008	0.000
MET31 (H)	13	3021	0.0000	0.0021	0.000
MET4 (H)	5	3010	0.0000	0.0008	0.000
MSN2 (H)	17	3021	0.0000	0.0028	0.000
OPI1 (H)	80	3025	0.0000	0.0131	0.000
PDR3 (H)	17	3023	0.0000	0.0028	0.000
PHO2 (H)	4	3020	0.0000	0.0007	0.000
RLM1 (H)	53	3020	0.0000	0.0087	0.000
RLR1 (H)	39	3020	0.0000	0.0064	0.000
RPN4 (H)	38	3023	0.0000	0.0063	0.000
SIG1 (H)	23	3014	0.0000	0.0038	0.000
SNT2 (H)	26	3021	0.0000	0.0043	0.000
SPT2 (H)	46	3020	0.0000	0.0076	0.000
STB5 (H)	12	3021	0.0000	0.0020	0.000
STP1 (H)	4	3014	0.0000	0.0007	0.000
SUT1 (H)	8	3020	0.0000	0.0013	0.000
THI2 (H)	8	3014	0.0000	0.0013	0.000
RPN4 (M)	29	3021	0.0000	0.0048	0.000
PUT3 (M)	3	3021	0.0000	0.0005	0.000
GCR1 (M)	11	3023	0.0000	0.0018	0.000
HAP1 (M)	24	3020	0.0000	0.0040	0.000
ABF1 (M)	38	3010	0.0000	0.0063	0.000
YRR1 (M)	13	3020	0.0000	0.0022	0.000
STB5 (M)	12	3020	0.0000	0.0020	0.000
ROX1 (M)	30	3021	0.0000	0.0050	0.000
YDR520C (M)	6	3021	0.0000	0.0010	0.000

Continued on next page

TF (Study)	Total	Prom	M_{Obs}	M_{Exp}	$M_{\text{Obs}}/M_{\text{Exp}}$
RLR1 (M)	2	3010	0.0000	0.0003	0.000
CAD1 (M)	11	3020	0.0000	0.0018	0.000
MIG1 (M)	19	3020	0.0000	0.0031	0.000
GCN4 (M)	39	3023	0.0000	0.0064	0.000
YAP1 (M)	7	3021	0.0000	0.0012	0.000
STP4 (M)	2	3007	0.0000	0.0003	0.000
AZF1 (M)	3	2996	0.0000	0.0005	0.000
MET32 (M)	30	3021	0.0000	0.0050	0.000
SFL1 (M)	19	3023	0.0000	0.0031	0.000
RAP1 (M)	54	3020	0.0000	0.0089	0.000
