# Supplementary material for: Redundancy and the Evolution of Cis-Regulatory Element Multiplicity
Source: PLoS Comput Biol. 2010 Jul 8;6(7):e1000848. doi: 10.1371/journal.pcbi.1000848 (PMC2900288; doi:10.1371/journal.pcbi.1000848)
Supplement: Table S4 — Matrix of correlations between genomic features. Values are Spearman's rank correlation coefficients (ρ). Data used for the cluster analysis in Figure 7. (0.04 MB PDF) [file pcbi.1000848.s009.pdf]

Table S4. Matrix of correlations between genomic features. Values are Spearman rank correlation coefficients (ρ). Data used for the cluster analysis in Figure 7.

	Len	Nucl	TATA	GC	DSB	Div	CO	GR	ER	BR	Essent	Dupl	K_a/K_s	DC	Noise	mRNA
Nucl	0.69															
TATA	0.20	0.13														
GC	0.28	0.23	0.23													
DSB	0.06	-0.03	0.16	0.37												
Div	-0.02	0.03	-0.13	-0.11	-0.27											
CO	0.21	0.13	0.10	0.15	0.19	-0.13										
GR	-0.19	-0.14	-0.27	-0.18	-0.11	0.09	-0.08									
ER	-0.20	-0.07	-0.23	-0.12	-0.10	0.11	-0.05	0.29								
BR	-0.09	-0.08	-0.14	-0.08	-0.02	-0.05	-0.06	0.20	0.13							
Ess	-0.09	-0.12	-0.13	-0.12	-0.07	0.04	-0.01	0.16	0.01	0.10						
Dupl	0.15	0.06	0.07	0.12	0.14	-0.13	0.03	-0.06	-0.05	0.01	-0.16					
K_a/K_s	-0.09	0.00	-0.14	-0.14	-0.17	0.23	-0.08	0.03	0.22	-0.04	-0.14	-0.02				
DC	0.10	-0.06	0.01	-0.00	0.02	-0.09	0.04	-0.02	-0.19	0.07	0.23	0.10	-0.33			
Noise	0.03	0.15	0.16	0.10	0.07	0.07	0.03	-0.08	0.15	-0.09	-0.21	0.01	0.23	-0.27		
mRNA	0.08	-0.12	0.07	0.08	0.11	-0.13	0.03	-0.01	-0.23	0.08	0.16	0.02	-0.44	0.36	-0.49	
Prot	0.06	-0.09	0.07	0.02	0.06	-0.09	0.04	-0.05	-0.30	0.01	0.17	-0.00	-0.45	0.36	-0.46	0.57

Legend: Len, promoter length; Nucl, proportion of sequence occupied by nucleosomes; TATA box presence/absence; GC content; DSB, frequency of meiotic DSBs; Div, proportion of nucleotide differences between *S. cerevisiae* and *S. paradoxus*; CO, number of crossovers; GR, genetic robustness; ER, environmental robustness; BR, genetic background robustness; Ess, essentiality; Dupl, presence of a gene duplicate elsewhere in the genome; DC, degree centrality; Noise, protein expression noise; mRNA abundance; Prot, protein abundance. See Protocol S1 for more details.
