# Supplementary material for: Redundancy and the Evolution of Cis-Regulatory Element Multiplicity
Source: PLoS Comput Biol. 2010 Jul 8;6(7):e1000848. doi: 10.1371/journal.pcbi.1000848 (PMC2900288; doi:10.1371/journal.pcbi.1000848)
Supplement: Table S5 — Association between multiplicity and GO slim terms from each domain. Significance levels after correction for multiple comparisons using the Holm method: *, P<0.01; **, P<0.001; ***, P<0.0001. (0.06 MB PDF) [file pcbi.1000848.s010.pdf]

Table S5 Association between multiplicity and GO slim terms from each domain. Significance levels after correction for multiple comparisons using the Holm method: *, $P < 0.01$; **, $P < 0.001$; ***, $P < 0.0001$.

ID	Description	Genes	Effect	(SE)	*Z*	*k*	
Cellular component							
**GO:0005886**	**plasma membrane**	281	0.058	0.0084	6.904	105	***
**GO:0005618**	**cell wall**	98	0.042	0.0066	6.365	74	***
**GO:0005624**	**membrane fraction**	196	0.040	0.0069	5.721	81	***
**GO:0005576**	**extracellular region**	25	0.029	0.0066	4.399	42	**
**GO:0016020**	**membrane**	1129	0.024	0.0058	4.082	128	*
GO:0005840	ribosome	338	0.013	0.0060	2.131	63	
GO:0005694	chromosome	337	0.010	0.0065	1.576	77	
GO:0005933	cellular bud	177	0.010	0.0064	1.541	64	
GO:0005815	microtubule organizing center	71	0.010	0.0077	1.240	39	
GO:0005783	endoplasmic reticulum	366	0.006	0.0060	0.971	68	
GO:0005777	peroxisome	62	0.007	0.0068	0.957	24	
GO:0005773	vacuole	208	0.005	0.0078	0.693	71	
GO:0005938	cell cortex	118	0.000	0.0061	0.043	53	
GO:0005739	mitochondrion	1051	–0.000	0.0058	–0.025	120	
GO:0030427	site of polarized growth	221	–0.002	0.0068	–0.331	70	
GO:0005730	nucleolus	233	–0.004	0.0100	–0.351	47	
GO:0005856	cytoskeleton	207	–0.003	0.0061	–0.485	65	
GO:0005740	mitochondrial envelope	305	–0.006	0.0062	–1.011	74	
GO:0005794	Golgi apparatus	182	–0.007	0.0065	–1.121	48	
GO:0016023	cytoplasmic membrane-bounded vesicle	104	–0.010	0.0069	–1.404	40	
GO:0005634	nucleus	1868	–0.010	0.0071	–1.441	126	
GO:0012505	endomembrane system	322	–0.014	0.0060	–2.252	63	
GO:0005575	cellular component	1576	–0.013	0.0059	–2.255	113	
GO:0005737	cytoplasm	3443	–0.019	0.0058	–3.263	149	
Molecular function							
**GO:0005215**	**transporter activity**	368	0.040	0.0062	6.385	102	***
**GO:0030528**	**transcription regulator activity**	317	0.030	0.0069	4.328	88	*
**GO:0003677**	**DNA binding**	332	0.030	0.0070	4.246	81	*
GO:0016874	ligase activity	169	0.019	0.0064	2.959	64	

Continued on next page

ID	Description	Genes	Effect	(SE)	Z	k	
GO:0016829	lyase activity	84	0.017	0.0063	2.735	52	
GO:0016491	oxidoreductase activity	267	0.015	0.0060	2.581	91	
GO:0005198	structural molecule activity	334	0.013	0.0062	2.089	65	
GO:0016779	nucleotidyltransferase activity	67	0.016	0.0079	2.073	32	
GO:0030234	enzyme regulator activity	208	0.012	0.0060	2.059	63	
GO:0003774	motor activity	16	0.013	0.0075	1.724	16	
GO:0016740	transferase activity	674	0.007	0.0059	1.188	104	
GO:0004386	helicase activity	80	0.008	0.0089	0.943	24	
GO:0004721	phosphoprotein phosphatase activity	48	0.006	0.0068	0.935	31	
GO:0016853	isomerase activity	57	0.004	0.0067	0.601	30	
GO:0008233	peptidase activity	110	0.002	0.0063	0.361	47	
GO:0045182	translation regulator activity	9	0.002	0.0096	0.220	7	
GO:0004871	signal transducer activity	44	0.000	0.0065	0.036	37	
GO:0004672	protein kinase activity	128	–0.001	0.0062	–0.135	55	
GO:0008289	lipid binding	71	–0.006	0.0065	–0.879	33	
GO:0003723	RNA binding	216	–0.009	0.0076	–1.160	44	
GO:0016787	hydrolase activity	784	–0.012	0.0071	–1.637	105	
GO:0005515	protein binding	529	–0.018	0.0060	–3.038	67	
**GO:0003674**	**molecular function (unknown)**	2778	–0.031	0.0060	–5.108	138	***
Biological process							
**GO:0044262**	**cellular carbohydrate metabolic process**	268	0.043	0.0064	6.770	94	***
**GO:0006091**	**generation of precursor metabolites and energy**	176	0.040	0.0076	5.339	76	*** ***
**GO:0006810**	**transport**	1016	0.024	0.0058	4.189	127	*
GO:0006457	protein folding	87	0.020	0.0071	2.820	38	
GO:0006519	cellular amino acid and derivative	250	0.018	0.0067	2.739	87	
	metabolic process	250	0.018	0.0067	2.739	87	
GO:0007124	pseudohyphal growth	65	0.017	0.0063	2.616	44	
GO:0006350	transcription	602	0.014	0.0059	2.428	89	
GO:0000746	conjugation	116	0.016	0.0066	2.351	53	
GO:0031505	fungal-type cell wall organization	127	0.013	0.0061	2.182	62	
GO:0007033	vacuole organization	55	0.012	0.0064	1.900	37	

Continued on next page

ID	Description	Genes	Effect	(SE)	*Z*	*k*
GO:0019725	cellular homeostasis	138	0.009	0.0060	1.482	60
GO:0045333	cellular respiration	92	0.011	0.0079	1.360	39
GO:0006412	translation	376	0.010	0.0075	1.301	68
GO:0044255	cellular lipid metabolic process	215	0.008	0.0065	1.276	58
GO:0006725	cellular aromatic compound	79	0.005	0.0063	0.816	40
	metabolic process	79	0.005	0.0063	0.816	40
GO:0030435	sporulation resulting in formation	132	0.006	0.0071	0.792	45
	of a cellular spore	132	0.006	0.0071	0.792	45
GO:0046483	heterocycle metabolic process	174	0.004	0.0062	0.716	61
GO:0007114	cell budding	89	0.004	0.0071	0.494	43
GO:0051186	cofactor metabolic process	164	0.003	0.0062	0.482	49
GO:0006766	vitamin metabolic process	61	0.002	0.0065	0.379	34
GO:0032196	transposition	45	0.002	0.0079	0.263	22
GO:0016050	vesicle organization	61	0.002	0.0071	0.263	26
GO:0016044	cellular membrane organization	271	0.001	0.0074	0.075	67
GO:0000910	cytokinesis	119	0.000	0.0062	0.040	50
GO:0007165	signal transduction	213	–0.000	0.0060	–0.067	71
GO:0070271	protein complex biogenesis	213	–0.001	0.0062	–0.155	52
GO:0006950	response to stress	575	–0.003	0.0081	–0.419	86
GO:0006259	DNA metabolic process	393	–0.005	0.0083	–0.547	63
GO:0006997	nucleus organization	64	–0.004	0.0067	–0.596	27
GO:0007031	peroxisome organization	53	–0.006	0.0079	–0.729	21
GO:0007059	chromosome segregation	140	–0.005	0.0064	–0.787	40
GO:0016070	RNA metabolic process	1066	–0.006	0.0071	–0.799	101
GO:0042254	ribosome biogenesis	316	–0.008	0.0092	–0.842	52
GO:0007049	cell cycle	532	–0.006	0.0060	–0.949	83
GO:0042221	response to chemical stimulus	416	–0.008	0.0073	–1.027	80
GO:0007126	meiosis	152	–0.008	0.0062	–1.217	49
GO:0032989	cellular component morphogenesis	170	–0.008	0.0061	–1.289	55
GO:0006464	protein modification process	535	–0.008	0.0059	–1.369	89
GO:0008150	biological process	2035	–0.010	0.0062	–1.563	130
GO:0016192	vesicle-mediated transport	353	–0.013	0.0070	–1.778	73
GO:0007005	mitochondrion organization	286	–0.013	0.0070	–1.814	53
GO:0044257	cellular protein catabolic process	184	–0.013	0.0062	–2.143	48

Continued on next page

ID	Description	Genes	Effect	(SE)	*Z*	*k*
GO:0007010	cytoskeleton organization	219	–0.014	0.0061	–2.373	61
GO:0051276	chromosome organization	382	–0.016	0.0061	–2.639	60
